# The Application of Functional Nanomaterials-Based Electrochemical Biosensors in Detecting Cancer Biomarkers: A Review

**DOI:** 10.3390/molecules30132708

**Published:** 2025-06-23

**Authors:** Meiyin Liu, Yuchen Song, Meiru Liu, Dongmei Deng, Wenjiao Zhang, Ting Wang, Liqiang Luo

**Affiliations:** 1 College of Sciences, Shanghai University, Shanghai 200444, China; liumeiyin@shu.edu.cn (M.L.); yuchensong@shu.edu.cn (Y.S.); liumeiru666888@shu.edu.cn (M.L.); dmdeng@shu.edu.cn (D.D.); zhangwenjiao@shu.edu.cn (W.Z.); 2 School of Health & Social Care, Shanghai Urban Construction Vocational College, Shanghai 201401, China

**Keywords:** electrochemical biosensors, functional nanomaterials, cancer biomarkers

## Abstract

Cancer remains a leading cause of morbidity and mortality worldwide. The timely and accurate detection of potential early cancer biomarkers is essential for early cancer diagnosis. In recent years, electrochemical biosensors have garnered significant attention due to their portability, cost-effectiveness, and ease of use. In addition, given their exceptional physicochemical properties, functional nanomaterials have found extensive applications in the field of electrochemical biosensing. This review provides an overview of the basic principles and types of electrochemical biosensors, the classification and properties of functional nanomaterials, and the application of functional nanomaterial-based electrochemical biosensors in the detection of cancer biomarkers. Finally, the prospects and remaining challenges of functional nanomaterial-based electrochemical biosensors in cancer diagnosis are discussed.

## 1. Introduction

Cancer is a disease with an extremely high death rate that raises significant concern [1,2]. Reports indicate that in 2020, more than 10 million individuals worldwide lost their lives due to cancer. Experts project that by 2040, this figure could rise to approximately 16 million [2]. However, late-stage diagnosis is an extremely important factor contributing to the high mortality rate, highlighting the critical importance of early detection and treatment in cancer [3]. Detecting cancer biomarkers is essential for understanding their physiological and biological functions in early disease diagnosis, monitoring, and prognosis [4,5]. Therefore, it is crucial to conduct early detection and screening of specific organs using specific biomarkers before tumor metastasis occurs [6].

In recent years, electrochemical biosensors have increasingly attracted attention for cancer biomarker detection due to their rapid response, minimal sample volume requirements, and high sensitivity [7,8,9]. Circulating cells, metabolites, protein antigens, carbohydrate antigens, and nucleic acids can serve as analyte targets for electrochemical biosensors.

Nanotechnology enables nanoscale customization of electrode surfaces, enhancing sensor performance. For example, functional nanomaterials possess large surface areas and excellent biocompatibility, serving as carriers for signaling molecules and facilitating signal transduction on electrode surfaces [10,11]. A variety of functional nanomaterials, including nanowires [12], nanotubes [13], nanocubes [14], and nanooctahedra [14,15], are commonly utilized in constructing diverse sensors. The integration of nanotechnology and nanomedicine is increasingly gaining attention in the field of biosensing.

This review systematically summarizes the fundamental principles and classifications of electrochemical biosensors and outlines recent advances in the application of functional nanomaterials for the electrochemical detection of cancer biomarkers. Compared with existing reviews [16,17,18], this work presents three notable strengths: first, it provides a comprehensive overview of representative studies from the past decade that employ functional nanomaterials for cancer biomarker detection, reflecting the latest developments in the field. Second, it highlights the performance advantages of various types of functional nanomaterials and elucidates their roles in enhancing key sensor characteristics such as sensitivity and specificity. Third, it categorizes the application of nanomaterial-based electrochemical biosensors according to different cancer types, thereby improving the review’s relevance and practical value. In addition, the review offers an in-depth discussion on the future prospects and major challenges of using such biosensors for early cancer diagnosis.

## 2. Electrochemical Biosensors

Biosensors integrate biological recognition elements such as enzymes, antibodies, nucleic acids, and cells with traditional sensor technologies, advancing quickly into areas such as molecularly sensitive receptors, biomimetic sensors, and nanotechnology [19,20]. Based on the types of biorecognition elements, biosensors can be categorized into immunosensors, aptasensors, enzyme biosensors, and so on.

Electrochemical biosensors are a type of biosensor that transduces biological information into measurable electrical signals, which are appreciated for their portability, low cost, and ease of use [21]. These systems are typically composed of three essential components: (a) a recognition element that specifically binds to the analyte; (b) a signal transducer that converts this interaction into a measurable signal, during which the electrode serves as the core component for the generation and transmission of electrochemical signals [22]; and (c) an electronic unit responsible for data processing. The recognition element is crucial, significantly impacting the sensor’s sensitivity and specificity in detection [23]. In electrochemical biosensors, the interaction between the recognition elements and biomarkers yields detectable electrochemical signals. These signals, whether oxidation-reduction potentials, electron transfer rates, or surface conductivity, are directly proportional to the concentration of the analytes [24]. Antibodies, aptamers, enzymes, and DNA fragments are widely used as recognition molecules due to their excellent affinity and specificity [25]. This section will primarily introduce electrochemical immunosensors and electrochemical aptasensors.

### 2.1. Electrochemical Immunosensors

**Figure 1:**
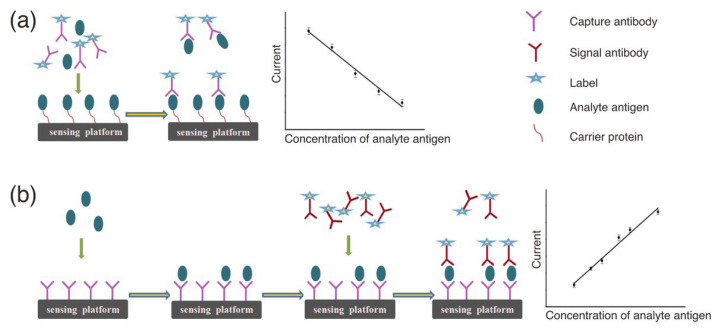
(**a**) Competitive immunosensors. (**b**) Non-competitive immunosensors [30]. Copyright (2016) Wiley.

Electrochemical immunosensors are electrochemical sensing platforms optimized for quantitative immunoassays through immune recognition reactions [26]. Antibodies, also referred to as immunoglobulins, are naturally occurring glycoproteins essential for the immune system in higher animals. Constructed from two identical short light chains and two identical long heavy chains, antibodies typically form heterodimers and are arranged in a Y-shaped structure [27]. Antibodies are the most classic recognition elements. The high specificity and selective immune recognition between antigens and antibodies facilitate the utilization of electrochemical immunosensors in analyzing complex biological matrices, including plasma, blood, and urine [28]. Typically, electrochemical immunosensors, which rely on the specific interaction between antigens and antibodies, can be divided into two categories: competitive immunosensors and non-competitive immunosensors [29]. Competitive immunoassay sensors typically operate on the principle where the target analyte competes with a signaling molecule-labeled antigen for recognition sites on the antibody surface (Figure 1a). Non-competitive immunosensors typically operate based on the principle that the analyte of interest undergoes immunological binding with two antibodies (the first antibody and the second antibody) (Figure 1b).

### 2.2. Electrochemical Aptasensors

**Figure 2:**
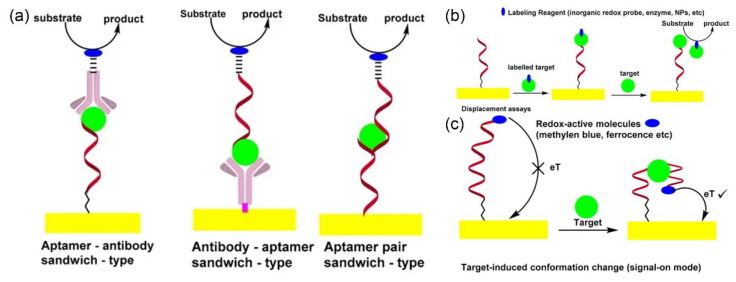
(**a**) Sandwich-type electrochemical aptasensors. (**b**) Displacement-type electrochemical aptasensors. (**c**) Folding-based electrochemical aptasensors [43]. Copyright (2021) MDPI.

Electrochemical aptamer sensors are a type of biosensor equipped with aptamer sequences that possess high affinity and serve as the biorecognition elements [31,32,33,34]. An aptamer refers to a single-stranded nucleic acid sequence (ssDNA or ssRNA) typically developed using systematic evolution of ligands and exponential enrichment for diagnostic purposes [35,36,37,38]. Aptamers and antibodies are generally utilized as recognition components in biosensors due to their ability to form highly accurate connections with target molecules. Factors such as immunogenicity, binding affinity, stability, and specificity are crucial considerations determining their applicability [39]. However, aptamers offer superior stability, smaller size, enhanced practicality, resistance to mutations (resulting in higher specificity), and are devoid of immunogenicity, in contrast to antibodies [40,41,42]. Based on different configurations, electrochemical aptasensors can also be categorized into sandwich-type electrochemical aptasensors, displacement-type electrochemical aptasensors, and folding-based electrochemical aptasensors [43] (Figure 2).

### 2.3. Discussion

Although electrochemical biosensors have attracted significant attention due to their high sensitivity, portability, and scalability, several critical challenges hinder their clinical translation. Firstly, most current systems lack real-time data processing capabilities, limiting their ability to provide rapid responses and continuous monitoring. Secondly, the fabrication procedures and operational protocols of these sensors are not yet standardized, resulting in poor reproducibility and limited comparability across studies. To advance the practical application of electrochemical biosensors, interdisciplinary strategies such as AI-assisted data analysis, microelectronic integration, and the establishment of standardized evaluation frameworks are urgently needed to enhance their real-time performance, reliability, and scalability.

## 3. Functional Nanomaterials

### 3.1. Carbon-Based Nanomaterials

Carbon-based materials, renowned for their diverse structural states, low cost, ease of production, rich surface properties, and outstanding electrochemical performance, have been extensively explored in the realm of electroanalysis [44]. Furthermore, nanostructured carbon-based materials are increasingly recognized as promising substitutes due to their distinctive structures and dimensions. Carbon nanotubes, graphene, carbon black, carbon quantum dots, and various composites derived from them stand out as well-established carbon-based nanomaterials.

**Figure 3:**
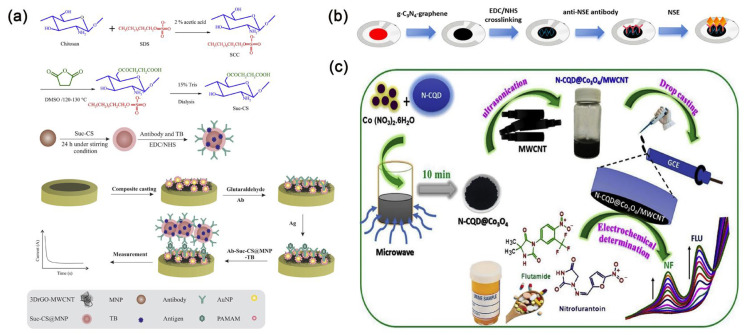
(**a**) Scheme of carbon nanotube-based electrochemical immunosensor for detecting CA 125 [49]. Copyright (2021) Elsevier. (**b**) Scheme of graphene-based electrochemical immunosensor for detecting NSE [52]. Copyright (2024) Nature. (**c**) Scheme of carbon quantum dot-based electrochemical immunosensor for detecting the antibiotic nitrofurantoin and the anticancer drug fluoxetine [61]. Copyright (2020) Elsevier.

Carbon nanotubes are generally classified according to their single-walled or multi-walled structures [45]. Single-walled carbon nanotubes are made up of individual graphene sheets, known for their small size and excellent conductivity. On the other hand, multi-walled carbon nanotubes consist of several layers of graphene sheets, with diameters that can reach up to 100 nanometers, offering greater surface area and customizable lengths. At the same time, they also possess extremely high conductivity. Carbon nanotubes are among the most promising candidates for biosensors [46]. Wang et al. [47] unveiled an implantable electrochemical sensor fabricated from functionalized multi-walled carbon nanotubes, which were intricately arranged into layered and helical fiber bundles. These bundles emulate the hierarchical architecture of muscles, boasting exceptional biocompatibility, flexibility, and robustness. This sensor can achieve monitoring of various biomarkers in the body. Cancer antigen 125 (CA 125) is one of the widely used biomarkers for diagnosing ovarian cancer, also known as the gold standard tumor marker for ovarian cancer [48]. Pakchin et al. [49] enhanced a glassy carbon electrode by incorporating a three-dimensional reduced graphene oxide-multiwalled carbon nanotube composite and immobilized PAMAM/AuNPs onto the electrode to create an electrochemical platform. The electrochemical immunosensor incorporated ortho-tolidine blue bonded to succinyl chitosan-coated magnetic nanoparticles for tracing, enabling ultra-sensitive detection of CA 125 (Figure 3a). This detection method exhibits a linear range from 0.0005 to 75 U mL^−1^, with a detection limit of approximately 6 μU mL^−1^. It is anticipated that this sensor will emerge as a reliable and effective detection tool in clinical applications.

In recent years, graphene-derived nanostructures have gained widespread applications due to their distinctive structural and physicochemical properties, including high conductivity, excellent flexibility, and strong adsorption capacity [50]. Today, graphene is gradually replacing carbon nanotubes in the development of biosensors. Researchers have shown keen interest in the biomedical applications of graphene and its derivative nanostructures. Andrews et al. [51] executed a study on the cardiovascular effects and acute pulmonary impact of inhaled graphene oxide on 14 healthy volunteers. They found that after inhaling highly pure graphene oxide nanosheets, the volunteers showed no significant changes in blood pressure, heart rate, inflammation markers, or lung function. This study confirms the feasibility of using graphene oxide materials in clinical settings. Zhang et al. [52] developed an electrochemical immunosensor to detect neuron-specific enolase (NSE), employing a screen-printed carbon electrode modified with a graphene–graphitic carbon nitride nanocomposite. The immunosensor exhibits a linear detection range spanning from 10 pg mL^−1^ to 100 ng mL^−1^, with an impressive detection limit of 3 pg mL^−1^ for NSE. This sensitivity holds significant promise for the early diagnosis of small cell lung cancer (SCLC) (Figure 3b). Additionally, in order to sensitively and specifically detect CA 125, Yan et al. [53] devised a novel paper-based electrochemical immunosensing platform utilizing a composite material of reduced graphene oxide/L-cysteine/gold nanoparticles. This method displays a detection linear range from 0.1 to 200 U mL^−1^ and an impressively low detection limit of 0.01 U mL^−1^. With its remarkable performance, it demonstrates substantial potential in early treatment strategy for ovarian cancer.

Carbon black, a commonly utilized carbon-based material, is highly favored by researchers for its significant surface area-to-volume ratio, ease of production, high conductivity, high porosity, and exceptional solvent dispersibility [54]. The composite of carbon black and polymer finds widespread application in fabricating electrochemical sensors due to its capacity to enhance electrode performance. Ibanez-Redin et al. [55] innovated an electrochemical sensing platform for CA 19-9 detection, characterized by its affordability and high sensitivity. This platform employs a screen-printed carbon electrode that is covered with carbon black and coated with a polymer electrolyte film. With a striking detection limit of 0.07 U mL^−1^, this material showcases promising potential for developing other biosensors.

Additionally, quantum dots possess adjustable colors, exceptional stability, narrow and symmetrical emission, broad and strong absorption, and solution processability, thus garnering significant attention from researchers. Building upon these advantages, doped quantum dots also exhibit reduced cytotoxicity and extended doping emission lifetimes [56]. Newly developed carbon-based nanomaterials, such as carbon quantum dots and graphene quantum dots, have attracted considerable attention in the field of biosensing. This interest is due to their unique properties, including optical and electrochemical features that stem from their quantum confinement effects [57]. Consequently, researchers often incorporate them into nanocomposite materials to greatly enhance sensor detection limits [58]. Carbon quantum dots also exhibit lower cytotoxicity and exceptional water dispersibility, thus playing a crucial role in the clinical medical field and gradually supplanting semiconductor quantum dots. On the other hand, transition metal oxides with nanostructures are often chosen as electrode modifiers [59]. It is found that electrode materials made by loading nitrogen-doped carbon quantum dots onto metal oxides can enhance charge transfer at the electrode/electrolyte interface [60]. For example, Muthusankar et al. [61] used a microwave synthesis method to load Co_3_O_4_ with nitrogen-doped carbon quantum dots and to hybridize them with multi-walled carbon nanotubes, creating a stable nanocomposite material for modifying glassy carbon electrodes. This material was used to fabricate an electrochemical sensor capable of detecting the antibiotic nitrofurantoin and the anticancer drug fluoxetine (Figure 3c). The sensor exhibits determination ranges of 0.05–1220 μM and 0.05–590 μM, and detection limits of 0.044 μM and 0.0169 μM, respectively, demonstrating satisfactory detection performance. Chowdhury et al. [62] devised an electrochemical sensor for detecting hepatitis E virus, employing antibodies coupled with nitrogen and sulfur co-doped graphene quantum dots alongside gold-embedded polyaniline nanowires. This sensor exhibits outstanding specificity and high sensitivity.

### 3.2. Metallic Nanomaterials

**Figure 4:**
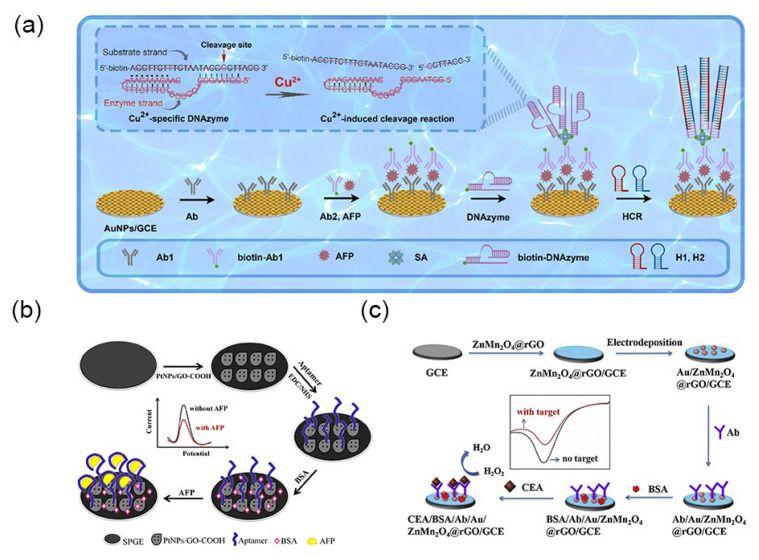
(**a**) Scheme of an electrochemical immunosensing platform based on gold nanoparticles for detecting AFP [71]. Copyright (2024) Elsevier. (**b**) Scheme of platinum nanoparticle-based electrochemical aptasensor for detecting AFP [72]. Copyright (2021) Nature. (**c**) Scheme of an amperometric immunosensor for detecting CEA [73]. Copyright (2022) Elsevier.

Gold nanoparticles have garnered widespread attention due to their simple preparation, high surface area ratio, quantum size effect and excellent cell compatibility [63,64]. Gold nanoparticles demonstrate unique plasmonic properties, catalytic capabilities, and electrochemical characteristics [65]. They are utilized to enhance surface plasmon resonance immunoassays [66], amplify optical or electrochemical signals [67], and play a crucial role in impedance immunosensors [68]. Yang et al. [69] engineered a novel electrochemical biosensing platform by decorating gold nanoparticles onto two-dimensional MXene nanomaterials. This innovative platform demonstrates high sensitivity and selectivity in detecting CEA. Furthermore, gold nanoparticles can be linked with biomolecules while preserving the biochemical activity of the labeled biomolecules [11]. Gold nanoparticles exhibit excellent biocompatibility and a narrow size distribution, rendering them ideal carriers for capture probes [70]. Recently, Chen et al. [71] developed an acutely sensitive electrochemical immunosensor for AFP detection (Figure 4a), employing a glassy carbon electrode enhanced with gold nanoparticles and incorporating an innovative cascade signal amplification approach. Under optimal conditions, the sensor exhibits a broad linear detection range from 0.001 to 100 ng mL^−1^ (R^2^ = 0.990), with an outstanding detection limit of 15.8 fg mL^−1^.

In addition to gold nanoparticles, other metallic nanoparticles, distinguished by their unique structures and exceptional electrochemical properties, are extensively employed in the design and fabrication of electrochemical biosensors. For example, Upan et al. [72] developed an electrochemical aptamer sensor for AFP detection, using platinum nanoparticles decorated on carboxylated graphene oxide. This design enhances the sensor’s surface area and increases the quantity of immobilized aptamers by incorporating COOH groups into graphene oxide (Figure 4b). This aptamer sensor is characterized by simple preparation and high sensitivity, with a linear range of 3.0–30 ng mL^−1^ and a low detection limit of 1.22 ng mL^−1^. However, compared to other metal nanoparticles, gold nanoparticles are often preferred by researchers for enhancing the effectiveness of immunoassays due to their strong conjugation capability, sensitive plasmon changes, and vivid colors.

Metallic compounds are also one of the key focuses of researchers. Fan et al. [73] developed a highly responsive and selective amperometric immunosensor for the detection of CEA (Figure 4c), utilizing a ZnMn_2_O_4_@reduced graphene oxide composite. The immunosensor demonstrates an excellent linear detection range from 0.01 to 50 ng mL^−1^ (R^2^ = 0.9988), with an impressive detection limit of 1.93 pg mL^−1^. Furthermore, Liu et al. [74] designed an electrochemical immunosensor based on cellulose nanofibrils/polydopamine/Cu-Ag nanocomposite, achieving ultra-sensitive detection of AFP.

### 3.3. Magnetic Nanomaterials

**Figure 5:**
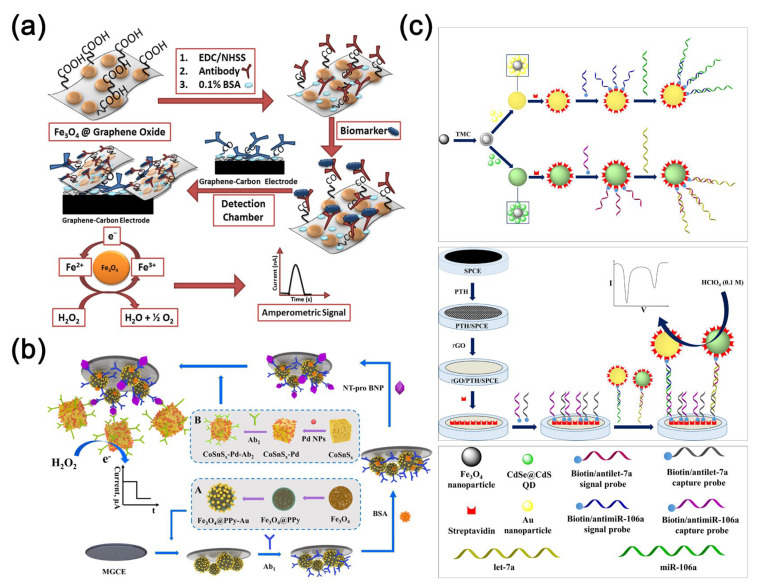
(**a**) Scheme of a mediator-free electrochemical detection platform based on magnetic composites (Fe_3_O_4_@reduced graphene oxide) for detecting prostate-specific antigen and prostate-specific membrane antigen [76]. Copyright (2017) Elsevier. (**b**) Scheme of an electrochemical sensing platform based on a magnetically controlled glassy carbon electrode for detecting N-terminal prohormone [77]. Copyright (2019) Elsevier. (**c**) Scheme of a dual-signal labeled electrochemical microRNA nanobiosensor based on Au/TMC/Fe_3_O_4_ and CdSe@CdS/TMC/Fe_3_O_4_ nanocomposites, capable of simultaneously detecting microRNA-106a and let-7a [80]. Copyright (2018) Elsevier.

Magnetic nanomaterials have captured considerable attention among researchers in the field of biosensing and analysis, owing to their distinctive properties encompassing magnetism, optics, and electrochemistry [75]. In previous studies, researchers have synthesized a variety of magnetic nanoparticles, including iron, cobalt, nickel, manganese oxides, and composite nanomaterials incorporating iron oxides [27]. The utilization of magnetic nanoparticles in analytical applications is primarily based on three strategies: Firstly, analyte preconcentration is achieved through the interaction of magnetic nanoparticles with analytes or by adsorbing immunocomplexes onto sensor surfaces using an external magnetic field. Secondly, sensors are enhanced by immobilizing magnetic nanoparticles onto them as functional materials. Lastly, magnetic nanoparticles serve as detection labels for analytes [27]. Sharafeldin et al. [76] innovatively developed a mediator-free electrochemical detection platform leveraging a magnetic composite material (Fe_3_O_4_@graphene oxide) achieved by integrating Fe_3_O_4_ nanoparticles onto graphene oxide nanosheets (Figure 5a). This sophisticated platform enables cost-effective and remarkably sensitive detection of prostate-specific antigen and prostate-specific membrane antigen. Remarkably, the method achieves an exceptional detection limit for prostate-specific antigen in serum, reaching as low as 15 fg mL^−1^, surpassing detection based solely on Fe_3_O_4_ by an astonishing 1000-fold improvement. Furthermore, detection of prostate-specific membrane antigen in serum exhibits an even lower detection limit of 4.8 fg mL^−1^. Li et al. [77] developed an electrochemical sensing platform for the detection of brain natriuretic peptide N-terminal prohormone using a magnetically controlled glassy carbon electrode, capable of immobilizing magnetic materials such as Fe_3_O_4_ under magnetic force (Figure 5b). The platform utilizes Fe_3_O_4_@PPyeAu as the substrate and amorphous bimetallic sulfide CoSnSxePd as the signal amplifier, with a low detection limit of 31.5 fg mL^−1^ and a wide linear detection range of 0.1 pg mL^−1^ to 50 ng mL^−1^. Zhang et al. [78] devised a disposable, cost-effective, miniature paper-based bipolar electrochemical luminescence aptasensor utilizing gold-coated Fe_3_O_4_ hybrid nanoparticles, exhibiting excellent efficacy in detecting CEA. As reported, the magnetic cores of nanocomposites are known to facilitate biomolecule separation during biosensing processes and enhance the surface conductivity of electrodes [79]. Daneshpour et al. [80] developed a novel electrochemical sensing platform employing dual-signal labeling for the concurrent detection of gastric cancer biomarkers microRNA-106a and let-7a. This platform is based on nanocomposites of Au/TMC/Fe_3_O_4_ and CdSe@CdS/TMC/Fe_3_O_4_ (Figure 5c). Moreover, the detection limits reached 0.02 fM and 0.06 fM, respectively.

### 3.4. Metal-Organic Frameworks (MOFs) Nanomaterials

MOFs are composed of metal ions and organic ligands. Incorporating functional groups (-NO_2_, -OH, -COOH, NH_2_, etc.), inorganic luminescent species, organic luminescent species, and nanoparticles into MOFs can enhance interactions such as hydrogen bonding, π interactions, and electrostatic interactions with guest molecules [81,82,83]. MOFs possess narrow size distribution, functional sites, and high porosity, enabling them to effectively capture a wide range of species within their pore spaces. This characteristic provides significant advantages in terms of regeneration, separation, storage, and the adjustability of framework structures. Researchers often optimize sensing performance by preloading various types of active substances into MOFs [84].

In practical applications, it is crucial to consider the reproducibility of manufactured MOF materials, as it determines their suitability for large-scale production. The repeatability of MOF-based sensors is influenced by the materials used, as well as the shape and structure of the pores. It has been reported that the repeatability of MOF-based sensors can be assessed by measuring the addition and release of biomarkers into MOFs, observing changes and recoveries in signals to evaluate the reusable capacity of MOFs [83].

**Figure 6:**
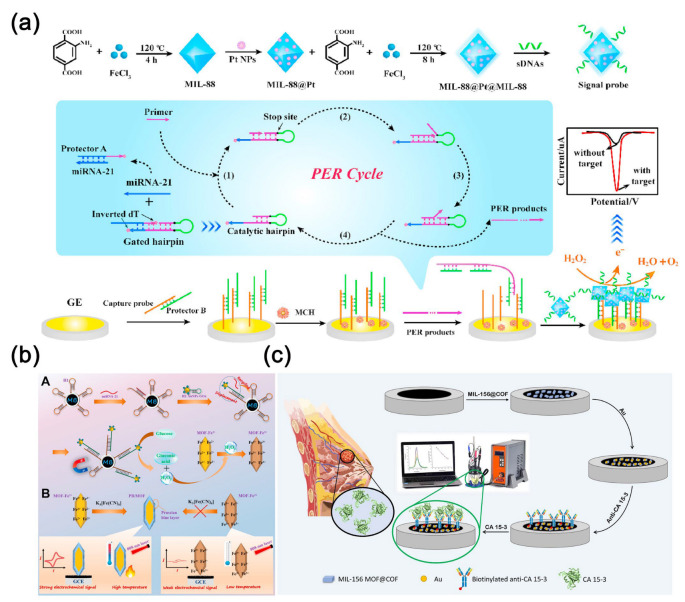
(**a**) Scheme of an electrochemical biosensing platform based on MOF@Pt@MOF nanozymes for detecting exosomal microRNA [86]. Copyright (2020) Elsevier. (**b**) Scheme of a photothermal and electrochemical sensing platform based on PB@MOF-Fe^2+^ for detecting microRNA-21 [89]. Copyright (2022) Elsevier. (**c**) Scheme of an immunosensor based on MOF-COF@Au for detecting CA15-3 [90]. Copyright (2024) Nature.

MOF-based nanoenzymes exhibit significantly enhanced catalytic activity compared to individual MOF structures and have been widely employed in sensing, catalysis, and cancer therapy [85]. Li et al. [86] proposed an electrochemical biosensing platform based on MOF@Pt@MOF nanoenzymes for the detection of exosome microRNA using a cascade primer exchange reaction. This platform demonstrates high selectivity, exhibiting promising prospects for early screening of tumor markers (Figure 6a). The detection linear range spans from 1 fM to 1 nM, with a detection limit of 0.29 fM. Combining MOF with graphene oxide can enhance both the electrochemical and mechanical properties. Hatami et al. [87] designed an innovative electrochemical sensing platform utilizing Cu MOF-reduced graphene oxide nanocomposite as the electrochemical probe and recognition platform for detecting MUC1 in human serum, achieving an impressive detection limit of 0.033 pM.

Accurate and sensitive detection of microRNA is crucial for the early diagnosis of non-small cell lung cancer (NSCLC) [88]. Tang et al. [89] innovatively combined the catalytic hairpin assembly amplification strategy with a novel biocatalysis-mediated MOF-to-Prussian blue (PB) transformation strategy to devise a photothermal and electrochemical sensing platform for microRNA-21 detection, utilizing PB@MOF-Fe^2+^ (Figure 6b). PB@MOF-Fe^2+^ facilitates temperature or electrochemical signal readout, while microRNA-21 triggers the CHA reaction on magnetic beads, capturing numerous glucose oxidase tags. Glucose oxidase catalyzes H_2_O_2_ generation from glucose, oxidizing Fe^2+^ to Fe^3+^ and inhibiting the MOF-to-PB transformation. Under optimal conditions, microRNA-21 detection limits were achieved at 0.3 fM and 0.32 fM for temperature and electrochemical readouts, respectively, underscoring remarkable detection sensitivity. Dezhakam et al. [90] developed an electrochemical biosensor based on the synergistic integration of MIL-156 MOF@COF and gold nanoparticles for the highly sensitive detection of the breast cancer biomarker CA15-3 (Figure 6c). The MIL-156 MOF was encapsulated by a COF to form a core-shell structure, which significantly increased the specific surface area and porosity of the material, while also enhancing the functionalization capability of the sensing interface. Co-modification of the electrode with the MOF@COF nanocomposite and AuNPs markedly improved the electrical conductivity, and the AuNPs provided efficient anchoring sites for antibody immobilization due to their strong affinity for amine groups. The combined effects of these nanomaterials effectively promoted electron transfer and biomolecular recognition, enabling a well-defined linear response in the concentration range of 30–100 nU mL^−1^, with a detection limit as low as 2.6 nU mL^−1^.

### 3.5. Discussion

In summary, various types of nanomaterials distinctly influence the key characteristics of electrochemical biosensors, such as sensitivity, selectivity, detection time, biocompatibility, and fabrication complexity (Table 1). Carbon-based nanomaterials have been extensively utilized in electrochemical biosensors owing to their excellent electrical properties, large specific surface area, and favorable biocompatibility. These features facilitate efficient electron transfer and stable signal output, as well as promote the immobilization of biomolecules, thereby enhancing sensor sensitivity and detection stability. Gold nanoparticles, characterized by unique plasmonic properties, superior biocompatibility, and strong biomolecular conjugation capabilities, are widely employed for signal amplification and labeling, significantly improving the sensitivity and selectivity of immunoassays. In comparison, other metallic nanoparticles, such as platinum nanoparticles, when decorated on functionalized supports, increase the active surface area and loading capacity of bioreceptors, thus enhancing electrochemical responses. However, their overall ease of use and recognition performance are generally inferior to those of gold nanoparticles. Magnetic nanomaterials leverage their magnetic responsiveness to enable rapid capture and separation of target analytes, substantially reducing detection time while improving sensitivity and selectivity. Moreover, their integration with electrode materials enhances electrical conductivity and overall sensor performance. MOFs exhibit highly porous structures and abundant functional sites, enabling efficient target molecule capture and signal amplification, with the added benefit of good reusability, although their synthesis procedures tend to be more complex. MOF-based nanozymes, which combine nanostructures with enzyme-mimicking catalytic functions, markedly amplify sensing signals, facilitating ultra-low concentration biomarker detection. The combination of MOFs with graphene oxide or magnetic nanoparticles further enhances mechanical strength and electrochemical properties, expanding the scope of sensing applications.

Overall, nanomaterials and nanotechnologies substantially improve the sensitivity, selectivity, and response speed of electrochemical biosensors by increasing surface area, catalytic activity, biomolecular recognition, and electron transfer efficiency. The diversity and tunability of nanomaterials provide a solid foundation for constructing high-performance, stable, and reusable sensing platforms, advancing bioanalytical techniques toward rapid, precise, and portable detection. This progress holds significant promise for clinical diagnostics and environmental monitoring applications.

## 4. The Application of Nanomaterial-Based Electrochemical Biosensors in Detecting Cancer Biomarkers

### 4.1. Breast Cancer

Breast cancer is the most common type of non-skin cancer found in women. To improve survival rates for breast cancer patients, accurate diagnosis and awareness of risks are particularly important. Early-stage breast cancer is often considered the ideal stage for treatment because of its high survival rate [91], contrasting sharply with the prognosis for late-stage cases. Thus, early diagnosis and prediction continue to be paramount for successful treatment [92]. However, breast cancer exhibits significant heterogeneity, posing challenges for conventional screening methods to discern diverse molecular features and risk factors [93]. In clinical practice, molecular subtyping is crucial for guiding targeted therapy and assessing prognosis. Breast cancer can be categorized into at least three subtypes according to the levels of progesterone receptor (PR), estrogen receptor (ER), and human epidermal growth factor receptor-2 (HER2): ER-positive (ER+)/PR-positive (PR+), HER2-positive (HER2+), and triple-negative subtypes [94].

**Figure 7:**
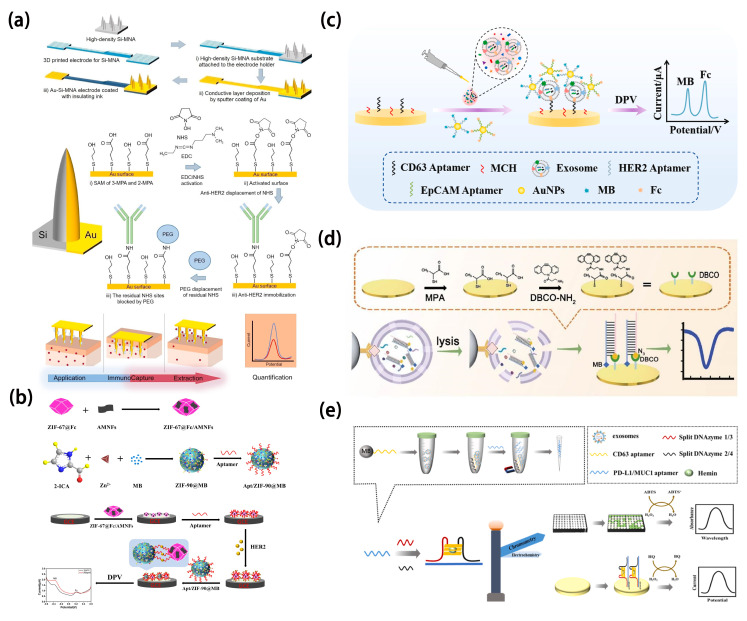
(**a**) Scheme of a microneedle array immunosensor detecting ErbB2 [97]. Copyright (2021) Elsevier. (**b**) Scheme of a ratiometric electrochemical impedance sensor for detecting HER2 [99]. Copyright (2022) Elsevier. (**c**) Scheme of breast cancer detection using an electrochemical strategy assisted by gold nanoparticles [102]. Copyright (2023) Elsevier. (**d**) Scheme of an electrochemical sensing platform capable of distinguishing and identifying exosomes from triple-negative breast cancer and estrogen receptor-positive breast cancer [111]. Copyright (2022) American Chemical Society. (**e**) Scheme of a dual-mode biosensing of surface proteins on breast cancer exosomes based on split horseradish peroxidase DNAzyme [112]. Copyright (2023) Elsevier.

In subtype detection of breast cancer, the patterns and levels of protein expression exhibit considerable diversity, emphasizing the significance of employing exosome-specific surface proteins as detection tools [95,96]. HER2 is primarily exhibited in the HER2-positive subtype of breast cancer. This subtype of breast cancer arises due to the overexpression of the HER2 gene or an increase in its copy number. Dervisevic et al. [97] extracted HER2 from a biomimetic gel mimicking the epidermal layer. They then utilized a high-density gold-coated silicon microneedle array to construct an electrochemical sensing platform for quantification. Their method exhibited a broad linear response (50–250 ng mL^−1^) and a low detection limit (25 ng mL^−1^) (Figure 7a).

In clinical applications, cost-effectiveness is also a critical consideration for researchers. Carvajal et al. [98] established a fully inkjet-printed electrochemical biosensing platform by employing gold and silver nanoparticle inks to fabricate integrated working, counter, and Ag/AgCl reference electrodes. This nanomaterial-enabled architecture substantially reduced production costs (below $0.25 per chip) while achieving rapid (15 min) and sensitive detection of the breast cancer biomarker HER2 in serum samples. Furthermore, a sandwich-type electrochemical immunoassay was implemented on the platform by introducing biotinylated antibodies, target HER2 proteins, and polymeric horseradish peroxidase conjugates into a microfluidic system. This work exemplifies the pivotal role of nanomaterials in advancing electrochemical biosensors by enhancing analytical performance, miniaturization, and clinical applicability for point-of-care diagnostics. The detection limit of the sensor is 12 pg mL^−1^. Xu et al. [99] devised a dual-signal ratiometric electrochemical aptasensor using ZIF-67 and ZIF-90 to capture HER2 through target-aptamer interaction and detect HER2 (Figure 7b). In this study, MOF materials such as ZIF-67 and ZIF-90 were introduced due to their high specific surface area, excellent electrical conductivity, and tunable porosity, which collectively enhanced the capture efficiency of HER2 and the signal amplification capability of the sensor. ZIF-90 was employed to encapsulate methylene blue (MB) as a signal probe, effectively minimizing interference from external environments. Meanwhile, ZIF-67 encapsulated ferrocene (Fc) and was combined with antimonene nanosheets (AMNFs), which significantly improved the adsorption of single-stranded DNA (ssDNA) and enabled signal modulation through target-induced desorption. Furthermore, the high hydrophilicity and biocompatibility of AMNFs contributed to the improved stability and molecular recognition performance of the sensing platform. The ratiometric detection strategy, based on the dual signal ratio (I_Fc_/I_MB_), effectively overcame the limitations of conventional single-signal electrochemical sensors, such as poor reproducibility and susceptibility to environmental interferences, thus greatly enhancing the accuracy, sensitivity, and reliability of detection. The sensor boasts a linear range of 0.5–1000 pg mL^−1^ and an impressively low detection limit of 155 fg mL^−1^. Furthermore, epidermal growth factor receptor [100] and intercellular adhesion molecule-1 [101] are typically co-expressed in the MDA-MB-231 cell line. Recently, Zhang et al. [102] developed an electrochemical aptasensor based on a multi-probe recognition strategy (Figure 7c). Utilizing SK-BR-3 as the model target and employing CD63, HER2, and EpCAM as the capture units, it effectively analyzed breast cancer exosomes. Gold nanoparticles functionalized with multiple probes enable efficient signal amplification and one-step multiplexed electrochemical detection of breast cancer exosomes, significantly enhancing the sensitivity and accuracy of exosomal biomarker detection in liquid biopsy. In addition to successfully distinguishing breast cancer exosomes from other exosomes, the sensor can further differentiate between HER2-positive and HER2-negative breast cancer exosomes, with a detection limit as low as 3.4 × 10^3^ particles mL^−1^. Moreover, it exhibits significant potential for exosome analysis in complex samples, promising valuable insights for detecting and assessing breast cancer. Han et al. [103] used MDA-MB-231 cells as a model to develop probes that specifically target surface biomarkers of the epidermal growth factor receptor and intercellular adhesion molecule-1. They devised an in-situ autonomous DNA assembly program combined with electrochemical techniques. This work achieves sensitive multiplexed detection of co-expressed therapeutic targets (EGFR and ICAM-1) on the surface of breast cancer cells through efficient loading and signal amplification mediated by quantum dots in an in situ automated DNA assembly reaction. This method facilitates the accurate identification of triple-negative breast cancer subtypes and introduces a novel testing approach for uncovering additional molecular subtypes.

MicroRNAs are small RNA sequences that are single-stranded and non-coding. They were initially discovered by Ambros et al. in 1993 [65] and can regulate up to 60% of protein-coding genes post-transcriptionally [104]. MicroRNAs play pivotal roles in multiple cellular processes, including both innate and adaptive immunity [105]. Pathogens can manipulate host microRNAs to undermine immune responses [106,107,108,109]. Hannafon et al. [110] utilized next-generation microRNA sequencing technology and qRT-PCR analysis to examine cellular and exosomal microRNAs in breast cancer cell lines. Their findings suggest that exosomal microRNA-1246 and microRNA-21 show promise as indicators of breast cancer. Cao et al. [111] introduced a biomimetic vesicle designed to simulate the breast cancer cell membrane and constructed an miRNA-375 detection platform, achieving a limit of detection as low as 557 particles mL^−1^. This enabled subtype-based molecular classification diagnosis of circulating exosomes (Figure 7d). These vesicles effectively targeted breast cancer exosomes, amplifying electrochemical signals for subtype identification. Magnetic nanoparticles (anti-CD44 functionalized immunomagnetic beads) are primarily used for the specific recognition and capture of biomimetic vesicles expressing CD44 on their surface, enabling efficient enrichment of breast cancer-derived exosomes and significantly enhancing the specificity and purity of the detection system. Validation experiments confirmed their accuracy and reliability, suggesting their potential in precise diagnosis and personalized treatment of breast cancer patients. Cheng et al. [112] developed a dual-modal sensor that combines naked-eye detection and electrochemical assays for detecting exosomal surface proteins PD-L1 and MUC1 in breast cancer (Figure 7e). In this study, magnetic nanoparticles (CD63 aptamer-functionalized magnetic beads) were employed for the specific capture of breast cancer-derived exosomes, enabling efficient enrichment and background elimination through magnetic separation, thereby enhancing the sensitivity and specificity of detection. Aptamers that are free from exosome binding can induce the formation of G-quadruplexes, serving as complete templates. This sensor distinguishes between non-metastatic individuals with metastatic breast cancer, and healthy individuals, using exosomal PD-L1 protein as the biomarker for MDA-MB-231 cells and exosomal MUC1 protein as the biomarker for MCF-7 cells. It boasts universality, simplicity, and cost-effectiveness as its distinctive features.

To simultaneously detect MCF7, MDA-MB-231, and SK-BR-3, Moura et al. [113] proposed a method that combined solid-phase preconcentration with immunomagnetic separation and electrochemical detection. They developed an electrochemical sensing platform in various formats, wherein magnetic particles were functionalized with antibodies against tetraspanins CD9, CD63, and CD81, along with specific cancer receptors (CD24, CD44, CD54, CD326, and CD340), to preconcentrate exosomes. Immunomagnetic separation was then conducted using anti-CD81-modified magnetic particles. Magnetic nanoparticles were employed as powerful preconcentration tools for the efficient isolation and enrichment of breast cancer cell-derived exosomes, thereby enhancing the sensitivity and specificity of subsequent electrochemical immunosensing for cancer diagnosis. CD24 and CD340 were selected as breast cancer-related biomarkers. The sensor exhibits a detection limit of 1.94 × 10^5^ exosomes μL^−1^ and 1.02 × 10^6^ exosomes μL^−1^. In addition, it demonstrates reliable results in distinguishing between non-cancerous individuals and breast cancer patients.

### 4.2. Cervical Cancer

Cervical cancer ranks among the most prevalent malignancies worldwide [114,115], with over half a million new diagnoses each year and more than 300,000 resulting deaths. Nearly all cases are attributed to high-risk strains of Human Papillomavirus (HPV), a disease that can largely be prevented. HPV-16 and HPV-18 account for 70% of cervical cancer cases worldwide. Together, eight specific types (HPV-16, HPV-18, HPV-33, HPV-45, HPV-31, HPV-58, HPV-52, and HPV-35) make up 90% of all cases [116]. In low-income countries, resource constraints often lead to a higher number of patients, as residents typically lack access to organized screening and HPV vaccination programs. Conversely, high-income countries typically offer more effective screening programs, resulting in a substantial decrease in the incidence and mortality rates of cervical cancer [117].

**Figure 8:**
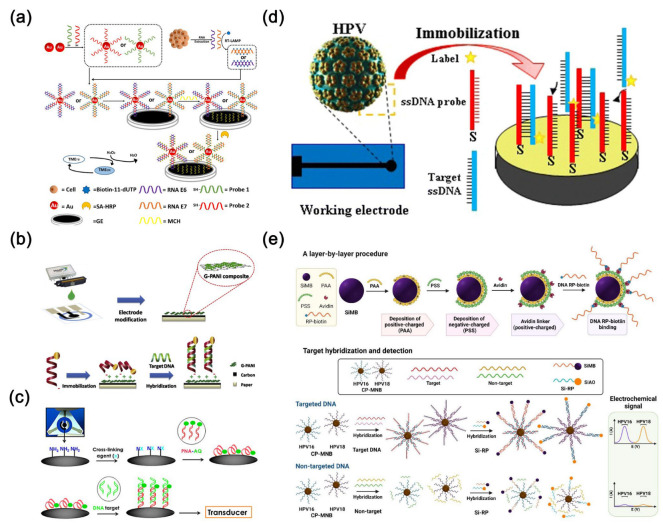
(**a**) Preparation process of an electrochemical biosensing platform based on a triple signal amplification strategy [118]. Copyright (2021) Elsevier. (**b**) Schematic diagram of electrode modification, immobilization, and hybridization steps in paper-based electrochemical DNA biosensor [119]. Copyright (2017) Elsevier. (**c**) Schematic diagram of immobilizing anthraquinone-labeled peptide nucleic acid probe on chitosan-screen-printed carbon electrode followed by hybridization with target DNA [120]. Copyright (2014) Elsevier. (**d**) Schematic diagram of a DNA biosensor for detecting HPV-18 based on ssDNA probe immobilization, DNA hybridization, and indicator intercalation detection [121]. Copyright (2020) Journal of Nanobiotechnology. (**e**) Schematic diagram of an electrochemical biosensor for detecting HPV-16 and HPV-18 [123]. Copyright (2022) MDPI.

HPV E6/E7 mRNA detection offers greater specificity and positive predictive value in screening compared to HPV DNA detection. For example, Yang et al. [118] developed an electrochemical biosensor with a triple signal amplification strategy. This biosensor utilizes gold nanoparticles in conjunction with reverse transcription loop-mediated isothermal amplification and a high-affinity biotin-streptavidin system to detect HPV 16 E6/E7 mRNA (Figure 8a). Gold nanoparticles serve as carriers for functionalizing specific DNA probes, enabling efficient capture and enrichment of target RNA, thereby enhancing detection sensitivity and specificity. Achieving a detection limit of 0.08 fM (approximately 100 copies), it shows promising potential for clinical applications. Teengam et al. [119] designed a paper-based electrochemical biosensor capable of detecting HPV type 16 DNA using PCR-amplified samples from the SiHa cell line (Figure 8b). This innovative sensor combines an anthraquinone-labeled pyrrolidinyl peptide nucleic acid probe with a graphene-polyaniline-modified electrode. Graphene was employed as an electrode modifier, and its high surface area and excellent conductivity enhanced the signal response and detection sensitivity of the paper-based electrochemical DNA biosensor. Produced using inkjet printing on paper, the sensor exhibits an impressive detection limit of 2.3 nM. Jampasa et al. [120] developed a highly sensitive electrochemical biosensor using an anthraquinone-labeled peptide nucleic acid probe that binds to a specific region of HPV type 16 (Figure 8c). This biosensor, based on a chitosan-modified disposable screen-printed carbon electrode, achieved a detection limit of 4 nM and a quantification limit of 14 nM for HPV type 16. It demonstrated a linear detection range from 0.02 to 12.0 mM and accurately differentiated between HPV-positive and HPV-negative samples. However, while successful in detecting HPV type 16 DNA in the SiHa cell line’s PCR-amplified samples, it could not detect the HPV-negative C-33A cell line.

Mahmoodi et al. [121] devised an electrochemical biosensor for sensitive and highly selective detection of HPV-18, utilizing a nanocomposite material composed of multi-walled carbon nanotubes and reduced graphene oxide. The sensor boasts remarkable specificity, speed, and accuracy, enabling the detection of minute quantities of analytes across a wide range with precision (Figure 8d). Reduced graphene oxide and multi-walled carbon nanotubes are electrodeposited onto the carbon electrode surface to form a nanocomposite with high conductivity and large surface area, enhancing electron transfer efficiency and signal response; subsequently, gold nanoparticles are drop-cast and functionalized with L-cysteine to create a stable linkage layer that facilitates efficient immobilization of single-stranded DNA (ssDNA) probes. This modification improves hybridization between the probe and target DNA, thereby enhancing the sensitivity and selectivity of the biosensor. Furthermore, the E6 region can sustain expression in high-risk HPV types and is believed to be associated with cervical cancer progression. Kim et al. [122] designed a novel electrochemical DNA biosensor based on a gold nanoparticle-modified electrode. They tested three targets: complementary targets for generating positive results, non-complementary targets, and mismatched targets for generating negative results, aiming to detect the 30-mer E6 region modified with HPV-18 DNA. In this study, gold nanoparticles were used to modify interdigitated electrodes (IDEs), enhancing probe immobilization and signal transduction, thereby improving the sensitivity and specificity of the electrochemical DNA biosensor for HPV-18 detection. Chaibun et al. [123] developed an electrochemical biosensor capable of concurrently detecting HPV-16 and HPV-18 using a “two-in-one” detection approach (Figure 8e). This sensor platform features two distinct redox indicators and silicon nanoparticles loaded with magnetic bead capture probes specifically. In this biosensor, magnetic silica nanoparticles serve to specifically capture HPV DNA, enable magnetic separation, and enhance signal amplification, thereby improving the sensitivity and specificity of detection. With detection limits of 22 fM for HPV-16 and 20 fM for HPV-18 within a detection range from 1 fM to 1 µM, it holds promising potential for clinical experimentation and epidemiological research. Moreover, Civit et al. [124] developed an electrochemical gene sensor utilizing a blend of thiolated probes and bipodal thiols terminated with oligoethylene glycol. Gold nanomaterials are employed to enhance the electrode’s electrical conductivity and surface area, thereby improving the immobilization efficiency of DNA probes and the hybridization efficiency of target DNA, ultimately resulting in increased sensitivity and specificity of the electrochemical sensor. This sensor enables simultaneous detection of HPV-16, HPV-18, and HPV-45 DNA sequences, facilitating high-throughput screening of multiple high-risk DNAs.

### 4.3. Lung Cancer

Lung cancer is notably prevalent. The factors contributing to lung cancer include smoking, passive smoking, environmental pollution, and so on [125,126]. In the early stage, most lung cancer patients do not exhibit significant symptoms; noticeable symptoms typically emerge only in advanced stages. Hence, early detection and screening are crucial [127]. Subdividing lung cancer into different subtypes can help patients receive more precise drug treatments. There are numerous classification methods for lung cancer. Histologically, it can be categorized into SCLC and NSCLC [89,128].

**Figure 9:**
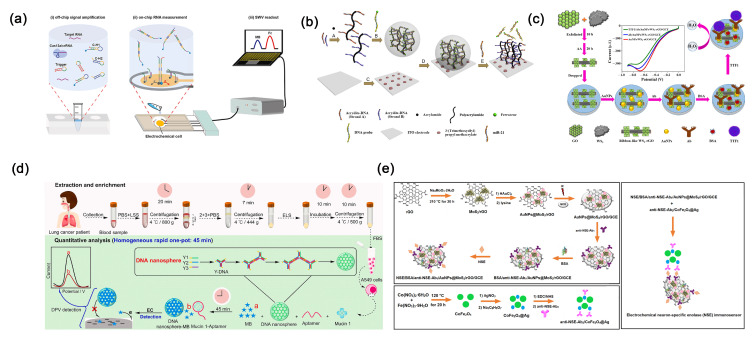
(**a**) The working principle of the Cas-CHDC-powered electrochemical RNA sensing technology chip [135]. Copyright (2021) Elsevier. (**b**) Scheme of an electrochemical DNA hydrogel biosensor based on mCHA signal coupled with gold nanoparticles/Mo_2_CNSs [139]. Copyright (2018) Elsevier. (**c**) Scheme of a label-free electrochemical immunosensor for detecting TTF-1 [143]. Copyright (2024) MDPI. (**d**) Scheme of a homogeneous electrochemical detection of circulating tumor cells [144]. Copyright (2024) Elsevier. (**e**) The working principle of the electrochemical NSE immunosensor [150]. Copyright (2022) Elsevier.

In incurable malignant tumors of the lungs, NSCLC accounts for approximately 80%. It is an aggressive cancer, primarily originating from the epithelial cell lining, insensitive to chemotherapy, and having a poor prognosis [129]. In late-stage NSCLC patients, symptoms of epidermal growth factor receptor mutation may arise. Hence, sensitive detection of epidermal growth factor receptor is pivotal for the treatment and prognosis of NSCLC patients [130]. Shoja et al. [131] developed a novel electrochemical gene biosensor for the sensitive and selective detection of epidermal growth factor receptor exon 2-point mutations. The nanocomposite material composed of reduced graphene oxide, ordered mesoporous carbon, and nickel-based conductive metal-organic polymer nanoparticles employed in this work enhances the electrochemical sensor’s performance by providing excellent electrical conductivity and a high specific surface area, which facilitate electron transfer and increase active sites. This effectively improves the electrode’s electrochemical reaction efficiency and DNA probe immobilization capacity, thereby significantly boosting the sensitivity and detection performance of the electrochemical biosensor. Under optimized conditions, the sensor exhibits long-term stability for 21 days, with a detection limit of 120 nM and a sensitivity of 0.0188 mA μM^−1^. Furthermore, microRNA-21 [132], Let-7, micoRNA-141 and microRNA-25 [133] are frequently utilized as diagnostic markers for lung cancer [134]. Sheng et al. [135] developed a strategy by combining the CRISPR/Cas13a system with the chemically hairpin DNA circuit (Figure 9a). This unique approach was integrated into an electrochemical biosensing platform, enabling highly selective and sensitive detection of six RNAs related to NSCLC in human serum, thereby distinguishing between NSCLC patients and those with benign lung diseases. The sensor can achieve a detection limit as low as 50 aM with a measurement volume of 10 μL in a short timeframe. Furthermore, Amineh et al. [136] devised an electrochemical nanosensor employing a hybrid of silver nanoparticles and single-walled carbon nanotubes for the detection of microRNA-25. In this work, the AgNPs/SWCNTs nanohybrid material facilitates the immobilization of single-stranded DNA probes on SWCNTs through π-π interactions, while the AgNPs serve as electrochemical signal generators; the synergistic effect of both components enhances the sensor’s sensitivity and selectivity, enabling efficient electrochemical detection of lung cancer-related miRNAs. The detection limit of this sensor is 3.13 × 10^−13^ M. In lung cancer, microRNA-21 emerges as a hallmark microRNA closely linked to cancer progression. Its abnormal expression fosters cell vitality, proliferation, and migration by targeting the B-cell translocation gene 2 while also suppressing apoptosis [137,138]. Liu et al. [139] devised an electrochemical sensor for detecting microRNA-21. They formed a hybrid DNA hydrogel by crosslinking ferrocene-labeled recognition probes with DNA grafted onto the main chain of polyacrylamide (Figure 9b). This hydrogel was then immobilized onto indium tin oxide electrode treated with 3-(trimethoxysilyl) propyl methacrylate. In addition, graphene oxide was used as a substrate material fixed on the silanized ITO electrode surface, providing a large specific surface area and abundant functional groups to facilitate the adsorption of DNA probes and enhance charge transfer. By employing the hybrid DNA hydrogel immobilized on the indium tin oxide/polyethylene terephthalate electrode, the biosensor was assembled. Hybridization of the target microRNA-21 with the recognition probe results in hydrogel dissolution, leading to loss of ferrocene labels and a change in current signal. The sensor achieves an ultra-low detection limit of 5 nM.

TTF-1, encoded by the NKX2-1 gene, regulates genes related to thyroid, lung, and forebrain differentiation. It serves as a marker for identifying differentiated thyroid or lung tissues [140,141]. In NSCLC, TTF-1 expression exhibits significant variability, which is coordinately regulated at the transcriptional level [142]. Wang et al. [143] developed a label-free electrochemical immunosensor using H_2_O_2_ as the electrochemical probe. This sensor leverages a nanocomposite of tungsten disulfide (WS_2_), known for its excellent catalytic activity and biocompatibility, and highly conductive reduced graphene oxide, enhancing the electrode’s electrochemical reactivity and signal amplification to achieve highly sensitive, label-free detection of the TTF1 biomarker (Figure 9c). It exhibits a linear detection range from 0.025 to 50 ng mL^−1^, with a low detection limit of 0.016 ng mL^−1^ (S/N = 3). A549 is a commonly used human NSCLC line, frequently employed in lung adenocarcinoma research. Recently, Liu et al. [144] introduced a streamlined, homogeneous electrochemical sensing platform for the rapid detection of circulating tumor cells in blood, with mucin 1 as the target marker. They employed Y-shaped DNA, created by hybridizing three distinct single-stranded DNA sequences, to self-assemble into nanospheres. This platform operates by disrupting the nanosphere structure when the mucin 1 antigen binds specifically to its aptamer. Given the heightened expression of mucin 1 on A549 cell surfaces, detecting mucin 1 indirectly enables the identification of A549 cells (Figure 9d). The platform offers linear detection ranges from 1 ag mL^−1^ to 1 fg mL^−1^ for mucin 1 and 1–100 cells mL^−1^ for A549 cells, respectively. Additionally, Wu et al. [145] proposed a one-step, homogeneous, and rapid electrochemical detection method for analyzing A549 cells.

In lung cancer cases, SCLC accounts for approximately 13–15% [146]. SCLC is distinguished by its rapid proliferation, dysregulated apoptosis, extensive vascularity, and early tendency for dissemination [147]. SCLC usually has the worst prognosis among lung cancer subtypes due to its low differentiation and brief clinical symptoms, often requiring multiple diagnostic evaluations [148].

NSE is a dimeric metalloenzyme found in central and neuroendocrine tissues, as well as peripheral neurons. Its overexpression in neuroendocrine tissues is associated with SCLC [149]. Karaman et al. [150] developed a highly selective and sensitive electrochemical sensor for the detection of NSE in plasma (Figure 9e), utilizing CoFe_2_O_4_@Ag nanocomposite and AuNPs@MoS_2_/rGO hybrid material. The sensor fully leverages the excellent conductivity, catalytic activity, and magnetic separation capability of nanomaterials, effectively enhancing electron transfer and specific recognition performance. In addition, the sensor demonstrates a detection limit as low as 3.0 fg mL^−1^, offering significant potential for the early diagnosis of SCLC.

### 4.4. Gastric Cancer

Despite a notable decline in the incidence of gastric cancer, it still ranks among the leading causes of cancer-related deaths worldwide. In some instances, the five-year survival rate for individuals diagnosed with gastric cancer remains quite low [151]. Several factors can contribute to the development of gastric cancer, including infection with Helicobacter pylori, high intake of nitrates and nitrites, smoking, and genetic predispositions [152]. Typically, gastric cancer can be classified into cardia gastric adenocarcinoma and non-cardia gastric adenocarcinoma [153]. Compared to advanced and late-stage gastric cancer, early-stage gastric cancer is highly treatable, making early detection of gastric cancer particularly important [154].

**Figure 10:**
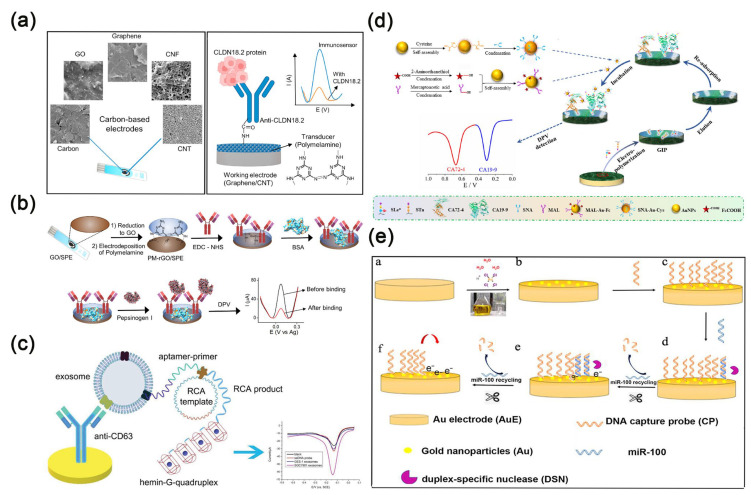
(**a**) Scheme of various carbon nanomaterial screen-printed electrodes and construction of polyamine-modified screen-printed electrodes for electrochemical immunosensors [156]. Copyright (2024) Elsevier. (**b**) Scheme of an electrochemical immunosensor constructed on the surface of reduced graphene oxide-polymelamine/screen-printed electrode [159]. Copyright (2024) Elsevier. (**c**) Scheme of a label-free electrochemical aptasensor for highly sensitive detection of exosomes [160]. Copyright (2019) Wiley. (**d**) Scheme of a glycan-imprinted electrochemical sensor capable of simultaneous detection of CA72-4 and CA19-9 [154]. Copyright (2023) Elsevier. (**e**) Scheme of a gold nanoparticle-based electrochemical sensor for detecting microRNA-100 [163]. Copyright (2021) Elsevier.

Claudin18.2, a member of the Claudin protein family, is a tight junction protein whose overexpression is indicative of gastric tumor growth and metastasis. This makes it a promising biomarker for the diagnosis of gastric cancer [155]. Kanagavalli et al. [156] developed the first Claudin18.2 immunosensor by electro-grafting an amine-rich conductive polymer, polyethyleneimine, onto various carbon nanomaterial screen-printed electrodes. Graphene and carbon nanotubes possess high electrical conductivity, abundant edge defects, and π-conjugated networks, which significantly enhance the formation efficiency of intermediate radicals and the electron transfer rate during the electropolymerization of melamine, thereby promoting the efficient deposition of the polymelamine layer and its excellent redox behavior. Therefore, comparative studies showed that polyethyleneimine deposited on graphene/screen-printed electrode and carbon nanotubes/screen-printed electrode exhibited the best redox behavior, with detection limits of 0.104 ng mL^−1^ and 7.9 pg mL^−1^, respectively (Figure 10a). Pepsinogen is a protein crucial for digestion and serves as a key marker for early-stage gastric cancer. Pepsinogen I, an immunogenic variant of pepsinogen, is predominantly secreted by mucous cells in the gastric fundus [157]. Evidence suggests that pepsinogen I concentrations in the serum of early gastric cancer patients are around 70.95 μg L^−1^ [158]. Recently, Kanagavalli et al. [159] employed cyclic voltammetry to deposit redox-active polyethyleneimine onto a reduced graphene oxide screen-printed electrode. They leverage the excellent electrical conductivity of reduced graphene oxide and the redox activity of polymelamine to construct a nanocomposite interface, which enhances electron transfer efficiency at the electrode and enables highly sensitive, label-free detection of pepsinogen I without the need for external redox probes, highlighting the pivotal role of nanomaterials in electrochemical biosensing. The cost-effective, disposable, and label-free electrochemical immunosensor enables sensitive detection of pepsinogen I over a clinically relevant range (0.01–200 ng mL^−1^), with a low detection limit of 9.1 pg mL^−1^. It demonstrates high specificity and excellent recovery performance in serum samples (Figure 10b).

Exosomes derived from gastric cancer cells significantly contribute to the pathological processes of tumors by enhancing chemoresistance, growth, migration, and invasion. These exosomes exhibit specific molecular characteristics depending on their cellular origin, making them promising potential tumor biomarkers. Huang et al. developed a label-free electrochemical aptasensor for detecting gastric cancer exosomes. This sensor employs gold nanoelectrodes to immobilize antibodies for exosome capture and utilizes hemin/G-quadruplex nanocomposites combined with rolling circle amplification to amplify the signal, thereby significantly enhancing the sensitivity and specificity of electrochemical exosome detection [160]. The aptasensor demonstrates high selectivity and sensitivity, with a linear detection range of 4.8 × 10^3^ to 4.8 × 10^6^ exosomes per milliliter and a detection limit of 9.54 × 10^2^ exosomes mL^−1^ (Figure 10c). In recent years, tumor markers, such as CA72-4 and CA19-9, have become crucial for diagnosing gastric cancer [161]. Simultaneous detection of multiple markers is recognized as essential for enhancing diagnostic accuracy [155]. Luo et al. [154] utilized glycosylation imprinting technology to develop an innovative electrochemical sensor capable of concurrently detecting CA72-4 and CA19-9. In this work, gold nanoparticles were employed as dopants and modification carriers, functionalized with cysteine and ferrocene to enable highly efficient electrochemical signal amplification and specific recognition of multiple tumor markers within the glycan-imprinted polymer sensor, thereby enhancing the sensitivity and selectivity of electrochemical sensing. Moreover, the sensor integrates lectins for specific binding to glycan chains, enabling precise identification of sialic acid in SleA, CA72-4, and CA19-9. Operating effectively across a concentration range of 0.005–200.0 U mL^−1^, this sensor offers accurate simultaneous detection of the gastric cancer biomarkers CA72-4 and CA19-9 (Figure 10d). Furthermore, Wu et al. [162] developed a portable sensor utilizing RNA aptamers, capable of sensitively detecting CA72-4.

MicroRNA-100 expression levels are significantly elevated in gastric cancer tissues compared to normal tissues. In response, Zhuang et al. [163] designed an electrochemical biosensor capable of sensitively, specifically, and rapidly detecting microRNA-100 levels in human serum, making it suitable for potential clinical applications in gastric cancer diagnosis. This sensor utilizes gold electrodes modified with gold nanoparticles and DNA capture probes. Gold nanoparticles enhance electrochemical sensing by providing a high surface area and excellent conductivity, enabling sensitive and specific detection of miR-100 in clinical samples through signal amplification and improved electrode interface properties. It operates effectively within a detection range from 100 aM to 10 pM, with a detection limit of 100 aM (Figure 10e). Recently, Zhu et al. designed an electrochemical sensor utilizing gold nanoparticles and DNA nanowires to detect sEV-microRNA-1246 in plasma samples from gastric cancer patients [164], achieving a detection limit of 5.24 aM. Gold nanoparticles serve as a robust high-surface-area scaffold supporting DNA nanowires, synergistically facilitating catalytic hairpin assembly (CHA) reactions, which significantly enhances the loading of electrochemical signaling molecules and improves the sensitivity and response efficiency of the sensor.

### 4.5. Other Cancers

**Figure 11:**
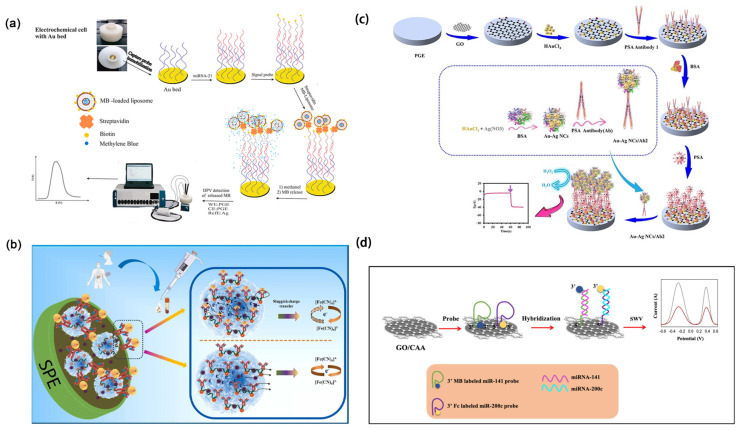
(**a**) Schematic diagram of an electrochemical biosensor for detecting colorectal cancer biomarkers [166]. Copyright (2025) Elsevier. (**b**) Schematic diagram of an electrochemical biosensor for detecting pancreatic cancer biomarkers [168]. Copyright (2024) Elsevier. (**c**) Schematic diagram of an electrochemical biosensor for detecting prostate cancer biomarkers [170]. Copyright (2025) Elsevier. (**d**) Schematic diagram of an electrochemical biosensor for detecting ovarian cancer biomarkers [173]. Copyright (2025) Elsevier.

Colorectal cancer ranks among the leading malignancies worldwide in both incidence and mortality, posing a significant threat to public health. Due to its often-asymptomatic nature in early stages, timely diagnosis is challenging, resulting in the majority of cases being detected at advanced stages, which markedly reduces treatment efficacy and patient survival. Therefore, enhancing early screening and diagnostic methods is crucial for improving prognosis and reducing the disease burden [165]. Norouzi et al. [166] utilized a gold nanoarray to enhance the electrode surface area, combined with methylene blue-encapsulated liposomes as signal amplification carriers, significantly improving the sensitivity and stability of the electrochemical biosensor for detecting the colorectal cancer biomarker microRNA-21 (Figure 11a). This work highlights the critical role of nanomaterials in signal amplification and sensor performance optimization. The sensor exhibited a wide dynamic detection range from 200 fM to 0.2 μM, with a detection limit as low as 85.0 fM.

Pancreatic cancer is a highly lethal malignancy characterized by nonspecific early symptoms and diagnostic challenges, often leading to late-stage detection and missed therapeutic opportunities. With rapid progression and aggressive behavior, current diagnostic methods lack sufficient sensitivity and specificity, contributing to a 5-year survival rate of only around 10%. As one of the leading causes of cancer-related deaths with a rising mortality rate, there is an urgent need for efficient, low-cost, and non-invasive early detection strategies to improve patient outcomes [167]. Huynh et al. [168] developed a nanomaterial-based electrochemical biosensor for the highly sensitive detection of the pancreatic cancer biomarker CA 19-9 (Figure 11b). In this work, erbium-doped graphene quantum dots (Er-GQDs) were decorated onto three-dimensional molybdenum disulfide nanoflowers to construct a composite probe material. The MoS_2_ nanoflowers provided a large surface area and excellent electrocatalytic activity, while the Er-GQDs served as functional anchoring sites to efficiently immobilize antibodies and enhance signal transduction. The resulting biosensor exhibited a wide dynamic detection range and ultra-low detection limits in real biological samples such as saliva and human serum, enabling non-invasive diagnosis. This work highlights the synergistic role of GQDs and MoS_2_ in enhancing biosensor performance for early cancer detection.

Prostate cancer is one of the most common malignant tumors in men. Due to its insidious early symptoms and rapid progression at later stages, it is often diagnosed at an advanced stage, severely affecting patient prognosis. Although PSA screening has improved early detection rates, its limited specificity can lead to overdiagnosis and unnecessary treatment, increasing the burden on patients. Therefore, the threat of prostate cancer lies not only in its lethality but also in the multiple challenges it poses to patients’ quality of life and healthcare systems [169]. Sadeghi et al. [170] developed a sandwich-type amperometric immunosensor for the ultra-sensitive detection of prostate-specific antigen (PSA), based on Au-Ag bimetallic nanoclusters and a reduced graphene oxide-gold nanoparticle composite (rGO-AuNPs) (Figure 11c). This work innovatively introduced Au-Ag NCs as signal amplification tags to enhance electrocatalytic activity, while the rGO-AuNPs substrate offered high conductivity and improved antibody immobilization and electron transfer efficiency. As a result, the sensor achieved outstanding sensitivity for PSA detection, with a wide linear range from 0.1 to 1.0 × 10^7^ pg mL^−1^, a detection limit of 30.0 fg mL^−1^, and a quantification limit of 100.0 fg mL^−1^, demonstrating strong potential for practical clinical applications.

Ovarian cancer poses a serious threat to women’s health, characterized by its high mortality rate and insidious early symptoms. Due to the lack of obvious clinical manifestations in the early stages, many patients are diagnosed only after the disease has progressed to an advanced stage, missing the optimal window for treatment and resulting in significantly reduced 5-year survival rates [171]. Current screening methods, such as CA-125 testing and transvaginal ultrasound, suffer from limited sensitivity and specificity, leading to both false negatives and false positives, which further complicate early detection. Statistically, ovarian cancer is the fifth leading cause of cancer-related deaths among women, with a notably higher incidence in older populations. Therefore, improving screening technologies and enhancing early diagnostic capabilities are crucial to reducing the mortality associated with ovarian cancer [172]. Kivrak et al. [173] innovatively developed a multiplexed electrochemical sensor platform based on carboxylated graphene oxide, which, due to its excellent surface functionalization capability, enhanced electron transfer properties, and stable biointerface, enabled ultra-sensitive and simultaneous detection of miR-141 and miR-200c (Figure 11d). The detection limits of the sensor were 0.029 pM and 0.026 pM, respectively. This high-performance nanobiosensing strategy offers great potential for the early screening of ovarian cancer.

### 4.6. Discussion

Based on the provided information, the application of nanomaterials in electrochemical biosensors for cancer detection demonstrates significant advantages (Table 2). Nanomaterials such as carboxylated graphene oxide and metallic nanoclusters enhance sensor performance through superior surface functionalization, efficient electron transfer, and stable biointerfaces, substantially improving sensitivity and detection limits. This enables early and simultaneous detection of multiple cancer-related biomarkers. Compared to conventional sensors, nanomaterial integration effectively amplifies signal response, enhances electron conductivity, and reduces background noise and false positives, thereby increasing detection accuracy and reliability. Moreover, the multifunctionality and tunability of nanomaterials allow for efficient, non-invasive, and portable detection across a wide range of cancers, including breast, lung, gastric, colorectal, pancreatic, prostate, and ovarian cancers. Overall, electrochemical biosensors integrated with nanomaterials not only optimize diagnostic performance but also advance early screening and precision medicine, highlighting their broad clinical application potential.

## 5. Conclusions and Perspective

Despite years of focused research, cancer continues to be a major cause of both global incidence and mortality. The persistent challenge of combating cancer emphasizes the crucial need for early and accurate diagnosis as well as effective risk evaluation. This review focuses on the research progress over the past decade in electrochemical biosensors based on functional nanomaterials for the detection of cancer biomarkers.

The integration of nanomaterials and nanotechnologies has significantly enhanced the sensitivity, selectivity, and response speed of electrochemical biosensors, primarily due to their high specific surface area, excellent catalytic activity, and superior electron transfer capability. The structural diversity and tunable functionalities of nanomaterials provide a solid foundation for constructing efficient, stable, and reusable sensing platforms, thereby facilitating the advancement of bioanalytical technologies in clinical and environmental diagnostics. However, several challenges remain in practical applications, including the controllable and scalable synthesis of nanomaterials, reproducibility issues, the stability and efficiency of biorecognition elements, and the absence of standardized evaluation systems. Future efforts should focus on developing green and efficient synthesis methods, integrating multifunctional sensing platforms, and combining artificial intelligence with portable devices to promote the development of intelligent and practical cancer diagnostic tools.

Additionally, it is widely recognized that cancers originating from different primary sites exhibit significant diversity. The integration of nanotechnology with electrochemical biosensors for site-specific cancer detection is now commonplace. However, substantial intra-tumor variability also exists within the same cancer type. In recent years, there has been increasing interest in classifying cancers into distinct subtypes based on molecular data and classification models. For example, molecular subtyping in breast cancer using ER, PR, and HER2 biomarkers has become standard in clinical practice. In addition, accurate identification of molecular subtypes remains a significant challenge in other types of cancers. The discovery and development of optimized biomarkers tailored to specific clinical needs pose significant challenges. Continued research in this field is expected to greatly enhance our capabilities in combating cancer.

## Figures and Tables

**Table 1:** Characteristics of various nanomaterials in electrochemical biosensor construction.

Nanomaterial Type	Key Features and Advantages	Impact on Sensor Performance	Limitations or Challenges	Feature	Limit of Detection	Ref.
Carbon-based nanomaterials	Excellent electrical conductivity, large surface area, and good biocompatibility.	Enhance electron transfer, stabilize signal output, promote biomolecule immobilization, and improve sensitivity and stability.	Material selection and functionalization need optimization.	Three-dimensional reduced graphene oxide-multiwalled carbon nanotube composite	CA 125 6 μU mL^−1^	[49]
				Graphene-graphitic carbon nitride nanocomposite	NSE 3 pg mL^−1^	[52]
				Reduced graphene oxide/L-cysteine/gold nanoparticles	CA 125 0.01 U mL^−1^	[53]
				Carbon black	CA 19-9 0.07 U mL^−1^	[55]
				Co_3_O_4_ with nitrogen-doped carbon quantum dots	Antibiotic nitrofurantoin and anticancer drug fluoxetine 0.044 μM and 0.0169 μM	[61]
Metallic nanomaterials	Unique plasmonic properties, superior biocompatibility, strong biomolecular conjugation ability, good catalytic activity, increased active surface area, and bioreceptor loading	Used for signal amplification and labeling, significantly improving sensitivity and selectivity, enhance electrochemical.	High cost, sensitive to storage and handling conditions, other metal nanoparticles (e.g., PtNPs) generally exhibit lower usability and recognition performance compared to AuNPs.	Gold nanoparticles	AFP 15.8 fg mL^−1^	[71]
				Platinum nanoparticles	AFP 1.22 ng mL^−1^	[72]
				ZnMn_2_O_4_	CEA 1.93 pg mL^−1^	[73]
Magnetic nanomaterials	Magnetic responsiveness enables rapid target capture/separation, and improved conductivity when integrated with electrodes.	Reduce detection time and enhance sensitivity and selectivity.	Complex structures and potential stability concerns.	Fe_3_O_4_	PSA and PSMA 15 fg mL^−1^ and 4.8 fg mL^−1^	[76]
				Fe_3_O_4_	Brain natriuretic peptide N-terminal prohormone 31.5 fg mL^−1^	[77]
				Fe_3_O_4_	MicroR-106a and let-7a 0.02 fM and 0.06 fM	[80]
Metal-organic frameworks (MOFs) nanomaterials	Highly porous structures, abundant functional sites, and reusable.	Efficient target capture, signal amplification, and expanded detection capabilities.	Complex synthesis procedures and reproducibility issues.	MIL-88@Pt@MIL-88	MicroRNA-21 0.29 fM	[86]
				Cu MOF	MUC1 0.033 pM	[87]
				Fe MOF	MicroRNA-21 (temperature and electrochemical readouts) 0.3 fM and 0.32 fM	[88]
				MOF-COF@Au	CA15-3 2.6 nU mL^−1^	[90]

**Table 2:** The application of nanomaterial-based electrochemical biosensors in the detection of various cancer biomarkers.

Cancer Type	Nanomaterial (Type)	Key Features and Advantages	Impact on Sensor	Limit of Detection	Ref.
Breast cancer	Gold and silver nanoparticles (metallic nanomaterials)	Reduce production costs.	below $0.25 per chip.	HER2 12 pg mL^−1^	[98]
	ZIF-67 and ZIF-90 (MOF nanomaterials)	High specific surface area, excellent electrical conductivity, and tunable porosity.	Improved the capture efficiency of HER2 and the signal amplification capability of the sensor.	HER2 155 fg mL^−1^	[99]
	Gold nanoparticles (metallic nanomaterials)	Excellent electrical conductivity, outstanding electrical conductivity, and good biocompatibility.	Perform efficient signal amplification to enhance sensitivity and accuracy.	HER2 3.4 × 10^3^ particles mL^−1^	[102]
	Anti-CD44 functionalized immunomagnetic beads (magnetic nanomaterials)	Specific recognition and capture of the target.	Efficient enrichment of breast cancer-derived exosomes and significantly enhancing the specificity and purity of the detection system.	MicroRNA-375 557 particles mL^−1^	[111]
	Magnetic particles (magnetic nanomaterials)	Specific recognition and capture of the target.	Enhance sensitivity and specificity.	CD24 and CD340 1.94 × 10^5^ exosomes μL^−1^ and 1.02 × 10^6^ exosomes μL^−1^	[113]
Cervical cancer	Gold nanoparticles (metallic nanomaterials)	Large specific surface area, serving as a carrier.	Efficient capture and enrichment of target RNA, thereby enhancing detection sensitivity and specificity.	HPV E6/E7 mRNA 0.08 fM	[118]
	Graphene (carbon-based nanomaterials)	High specific surface area and excellent electrical conductivity.	Enhance the signal response and detection sensitivity of the paper-based electrochemical DNA biosensor.	HPV type 16 DNA 2.3 nM	[119]
	Magnetic silica nanoparticles (magnetic nanomaterials)	Specific recognition and capture of the target.	Achieve signal amplification and enhance sensitivity and specificity.	HPV-16 and HPV-18 22 fM and 20 fM	[123]
Lung cancer	Reduced graphene oxide (Carbon-based nanomaterials)	Excellent electrical conductivity and high specific surface area.	Effectively improve the electrode’s electrochemical reaction efficiency and DNA probe immobilization capacity.	Receptor exon 2-point mutations 120 nM	[131]
	AgNPs/SWCNTs (metallic Nanomaterials and carbon-based nanomaterials)	Excellent electrical conductivity and high specific surface area.	The synergistic effect of both components enhances the sensor’s sensitivity and selectivity, enabling efficient electrochemical detection of lung cancer-related miRNAs.	MicroRNA-25 3.13 × 10^−13^ M	[136]
	Graphene oxide (carbon-based nanomaterials)	Large specific surface area and abundant functional groups	Facilitate the adsorption of DNA probes and enhance charge transfer.	MicroRNA-21 5 nM	[139]
	Reduced graphene oxide (carbon-based nanomaterials)	Highly conductive	Enhance the electrode’s electrochemical reactivity and signal amplification to achieve highly sensitive detection.	TTF-1 0.016 ng mL^−1^	[141]
Gastric cancer	Reduced graphene oxide (carbon-based nanomaterials)	Excellent electrical conductivity	Enhance electron transfer efficiency at the electrode and enable highly sensitivity.	Pepsinogen I 9.1 pg·mL^−1^	[159]
	Gold nanoparticles (metallic nanomaterials)	High surface area and excellent conductivity	Enhance the sensitivity and specificity of electrochemical exosome detection.	Gastric cancer exosomes 9.54 × 10^2^ exosomes mL^−1^	[160]
	Gold nanoparticles (metallic nanomaterials)	High surface area and excellent conductivity	Achieve signal amplification and enhance the sensitivity and specificity of target detection.	MicroRNA-100 100 aM	[163]
Colorectal cancer	Gold nanoparticles (metallic nanomaterials)	High surface area	Achieve signal amplification	MicroRNA-21 85.0 fM	[166]
Pancreatic cancer	Er-GQDs and MoS_2_ nanoflowers (carbon-based nanomaterials and metallic nanomaterials)	The MoS_2_ nanoflowers provide a large surface area and excellent electrocatalytic activity, while the Er-GQDs serve as functional anchoring sites.	Efficiently immobilize antibodies and enhance signal transduction.	CA 19-9 (0.18–2.95) × 10^−4^ U mL^−1^	[168]
Prostate cancer	Au–Ag bimetallic nanoclusters and a reduced graphene oxide–gold nanoparticle composite (carbon-based nanomaterials and metallic nanomaterials)	Au–Ag NCs as signal amplification tags, the rGO–AuNPs substrate offered high conductivity.	Enhance electrocatalytic activity, improve antibody immobilization and electron transfer efficiency.	PSA 30.0 fg/mL	[170]
Ovarian cancer	Carboxylated graphene oxide (carbon-based nanomaterials)	Excellent surface functionalization capability.	Enhanced electron transfer properties and stable biointerface.	MicroR-141 and microR-200c 0.029 pM and 0.026 pM	[173]

## References

[1] Mathur P., Sathishkumar K., Chaturvedi M., Das P., Sudarshan K.L., Santhappan S., Nallasamy V., John A., Narasimhan S., Roselind F.S. (2020). Cancer statistics, 2020: Report from national cancer registry programme, India. JCO Glob. Oncol..

[2] Joshi S., Kallappa S., Kumar P., Shukla S., Ghosh R. (2022). Simple diagnosis of cancer by detecting CEA and CYFRA 21-1 in saliva using electronic sensors. Sci. Rep..

[3] Ullah M.F., Aatif M. (2009). The footprints of cancer development: Cancer biomarkers. Cancer Treat. Rev..

[4] Ribeiro J.A., Sales M.G.F., Pereira C.M. (2022). Electrochemistry combined-surface plasmon resonance biosensors: A review. TrAC–Trends Anal. Chem..

[5] Hansson O. (2021). Biomarkers for neurodegenerative diseases. Nat. Med..

[6] Khan H., Shah M.R., Barek J., Malik M.I. (2023). Cancer biomarkers and their biosensors: A comprehensive review. TrAC–Trends Anal. Chem..

[7] Zhang Z., Li Q., Du X., Liu M. (2020). Application of electrochemical biosensors in tumor cell detection. Thorac. Cancer.

[8] Karimi-Maleh H., Khataee A., Karimi F., Baghayeri M., Fu L., Rouhi J., Karaman C., Karaman O., Boukherroub R. (2022). A green and sensitive guanine-based DNA biosensor for idarubicin anticancer monitoring in biological samples: A simple and fast strategy for control of health quality in chemotherapy procedure confirmed by docking investigation. Chemosphere.

[9] Karimi-Maleh H., Alizadeh M., Orooji Y., Karimi F., Baghayeri M., Rouhi J., Tajik S., Beitollahi H., Agarwal S., Gupta V.K. (2021). Guanine-based DNA biosensor amplified with Pt/SWCNTs nanocomposite as analytical tool for nanomolar determination of daunorubicin as an anticancer drug: A docking/experimental investigation. Ind. Eng. Chem. Res..

[10] Yang M., Tang J., Liu H., Zhang H., Zhang H. (2020). A novel demodulation method based on spectral clustering for phase-modulated signals interrupted by the plasma sheath channel. IEEE Trans. Plasma Sci..

[11] Marti A., Huskens J. (2022). Au nanoparticle-based amplified DNA detection on poly-L-lysine monolayer-functionalized electrodes. Nanomaterials.

[12] Gong S., Zhang X., Nguyen X.A., Shi Q., Lin F., Chauhan S., Ge Z., Cheng W. (2023). Hierarchically resistive skins as specific and multimetric on-throat wearable biosensors. Nat. Nanotechnol..

[13] Koman V.B., Bakh N.A., Jin X., Nguyen F.T., Son M., Kozawa D., Lee M.A., Bisker G., Dong J., Strano M.S. (2022). A wavelength-induced frequency filtering method for fluorescent nanosensors in vivo. Nat. Nanotechnol..

[14] Wu H.J., Henzie J., Lin W.C., Rhodes C., Li Z., Sartorel E., Thorner J., Yang P., Groves J.T. (2012). Membrane-protein binding measured with solution-phase plasmonic nanocube sensors. Nat. Methods.

[15] Han X., Li L., Wang C. (2012). Synthesis of tin dioxide nanooctahedra with exposed high-index 332 facets and enhanced selective gas sensing properties. Asial J. Chem..

[16] Malik S., Singh J., Goyat R., Saharan Y., Chaudhry V., Umar A., Ibrahim A.A., Akbar S., Ameen S., Baskoutas S. (2023). Nanomaterials-based biosensor and their applications: A review. Heliyon.

[17] Zhu C., Yang G., Li H., Du D., Lin Y. (2015). Electrochemical sensors and biosensors based on nanomaterials and nanostructures. Anal. Chem..

[18] Cho I.H., Kim D.H., Park S. (2020). Electrochemical biosensors: Perspective on functional nanomaterials for on site analysis. Biomater. Res..

[19] Liu Q., Wu C., Cai H., Hu N., Zhou J., Wang P. (2014). Cell-based biosensors and their application in biomedicine. Chem. Rev..

[20] Turner A.P. (2013). Biosensors: Sense and sensibility. Chem. Soc. Rev..

[21] Rotariu L., Lagarde F., Jaffrezic-Renault N., Bala C. (2016). Electrochemical biosensors for fast detection of food contaminants—Trends and perspective. TrAC–Trends Anal. Chem..

[22] Chen J., Lou Y., Sun L., Chia C.H., Nilghaz A., Tian J. (2025). Play on electrodes. ACS Sens..

[23] Bandodkar A.J., Wang J. (2014). Non-invasive wearable electrochemical sensors: A review. Trends Biotechnol..

[24] Hammond J.L., Formisano N., Estrela P., Carrara S., Tkac J. (2016). Electrochemical biosensors and nanobiosensors. Essays Biochem..

[25] Justino C.I.L., Freitas A.C., Pereira R., Duarte A.C., Rocha Santos T.A.P. (2015). Recent developments in recognition elements for chemical sensors and biosensors. TrAC–Trends Anal. Chem..

[26] Kim J., Park M. (2021). Recent progress in electrochemical immunosensors. Biosensors.

[27] Farka Z., Jurik T., Kovar D., Trnkova L., Skladal P. (2017). Nanoparticle-based immunochemical biosensors and assays: Recent advances and challenges. Chem. Rev..

[28] Chen H., Zhang J., Huang R., Wang D., Deng D., Zhang Q., Luo L. (2023). The applications of electrochemical immunosensors in the detection of disease biomarkers: A review. Molecules.

[29] Hua X., Zhou L., Feng L., Ding Y., Shi H., Wang L., Gee S.J., Hammock B.D., Wang M. (2015). Competitive and noncompetitive phage immunoassays for the determination of benzothiostrobin. Anal. Chim. Acta.

[30] Huo X., Liu X., Liu J., Sukumaran P., Alwarappan S., Wong D.K.Y. (2016). Strategic applications of nanomaterials as sensing platforms and signal amplification markers at electrochemical immunosensors. Electroanalysis.

[31] Negahdary M., Angnes L. (2023). Recent advances in electrochemical nanomaterial-based aptasensors for the detection of cancer biomarkers. Talanta.

[32] Chu T., Zhang C., Huang R., Zhang W., Deng D., Yan X., Zhang Q., Luo L. (2024). NiCo_2_S_4_ Nanosheets Supported on Cu_7_S_4_ Microcubes for the Electrochemical Detection of Glucose in Human Serum. ACS Appl. Nano Mater..

[33] Long Y., Zhao J., Ma W., He C., Pei W., Hou J., Hou C., Huo D. (2024). Fe single-atom carbon dots nanozyme collaborated with nucleic acid exonuclease III-driven DNA walker cascade amplification strategy for circulating tumor DNA detection. Anal. Chem..

[34] Li Y., Chen H., Huang R., Deng D., Yan X., Luo L. (2024). An origami microfluidic paper device based on core-shell Cu@Cu_2_S@N-doped carbon hollow nanocubes. Anal. Chim. Acta.

[35] Zhang Y., Yan X., Chen Y., Deng D., He H., Lei Y., Luo L. (2024). ZnO-CeO_2_ Hollow nanospheres for selective determination of dopamine and uric acid. Molecules.

[36] Dong J., Ouyang X., Huo B., Deng D., Yan X., Luo L. (2024). CuO/CoZn-layered double-hydroxide nanowires on carbon cloth as an enzyme-free H_2_O_2_ sensor. ACS Appl. Nano Mater..

[37] Chen Y., Wang H., Chen H., Song J., Deng D., Luo L. (2023). Synthesis of quaternary (Ni, Co, Cu)Se_2_ nanosheet arrays on carbon cloth for non-enzymatic glucose determination. Chemosensors.

[38] Rosch J.C., Balikov D.A., Gong F., Lippmann E.S. (2020). A systematic evolution of ligands by exponential enrichment workflow with consolidated counterselection to efficiently isolate high-affinity aptamers. Eng. Rep..

[39] Mikaeeli Kangarshahi B., Naghib S.M., Rabiee N. (2024). DNA/RNA-based electrochemical nanobiosensors for early detection of cancers. Crit. Rev. Clin. Lab. Sci..

[40] Negahdary M. (2020). Aptamers in nanostructure-based electrochemical biosensors for cardiac biomarkers and cancer biomarkers: A review. Biosens. Bioelectron..

[41] Negahdary M., Angnes L. (2022). Electrochemical aptamer-based nanobiosensors for diagnosing Alzheimer’s disease: A review. Mater. Sci. Eng. C-Mater. Biol. Appl..

[42] Atapour A., Khajehzadeh H., Shafie M., Abbasi M., Mosleh-Shirazi S., Kasaee S.R., Amani A.M. (2022). Gold nanoparticle-based aptasensors: A promising perspective for early-stage detection of cancer biomarkers. Mater. Today Commun..

[43] Abd-Ellatief R., Abd-Ellatief M.R. (2021). Electrochemical aptasensors: Current satus and future perspectives. Diagnostics.

[44] Zhang W., Zhu S., Luque R., Han S., Hu L., Xu G. (2016). Recent development of carbon electrode materials and their bioanalytical and environmental applications. Chem. Soc. Rev..

[45] Chen A., Chatterjee S. (2013). Nanomaterials based electrochemical sensors for biomedical applications. Chem. Soc. Rev..

[46] Zhang P., Zhu B., Du P., Travas-Sejdic J. (2024). Electrochemical and electrical biosensors for wearable and implantable electronics based on conducting polymers and carbon-based materials. Chem. Rev..

[47] Wang L., Xie S., Wang Z., Liu F., Yang Y., Tang C., Wu X., Liu P., Li Y., Saiyin H. (2020). Functionalized helical fibre bundles of carbon nanotubes as electrochemical sensors for long-term in vivo monitoring of multiple disease biomarkers. Nat. Biomed. Eng..

[48] Gupta D., Lis C.G. (2009). Role of CA125 in predicting ovarian cancer survival—A review of the epidemiological literature. J. Ovarian Res..

[49] Samadi Pakchin P., Fathi M., Ghanbari H., Saber R., Omidi Y. (2020). A novel electrochemical immunosensor for ultrasensitive detection of CA125 in ovarian cancer. Biosens. Bioelectron..

[50] Mukherjee S., Mukherjee A., Bytesnikova Z., Ashrafi A.M., Richtera L., Adam V. (2024). 2D graphene-based advanced nanoarchitectonics for electrochemical biosensors: Applications in cancer biomarker detection. Biosens. Bioelectron..

[51] Andrews J.P.M., Joshi S.S., Tzolos E., Syed M.B., Cuthbert H., Crica L.E., Lozano N., Okwelogu E., Raftis J.B., Bruce L. (2024). First-in-human controlled inhalation of thin graphene oxide nanosheets to study acute cardiorespiratory responses. Nat. Nanotechnol..

[52] Zhang J., Wei Z., Ttang Z., Li J.Y., An P., Zhang M., An H. (2024). Novel electrochemical platform based on C_3_N_4_-graphene composite for the detection of neuron-specific enolase as a biomarker for lung cancer. Sci. Rep..

[53] Fan Y., Shi S., Ma J., Guo Y. (2019). A paper-based electrochemical immunosensor with reduced graphene oxide/thionine/gold nanoparticles nanocomposites modification for the detection of cancer antigen 125. Biosens. Bioelectron..

[54] Silva T.A., Moraes F.C., Janegitz B.C., Fatibello-Filho O. (2017). Electrochemical biosensors based on nanostructured carbon black: A review. J. Nanomater..

[55] Ibáñez-Redín G., Materon E.M., Furuta R.H.M., Wilson D., do Nascimento G.F., Melendez M.E., Carvalho A.L., Reis R.M., Oliveira O.N., Gonçalves D. (2020). Screen-printed electrodes modified with carbon black and polyelectrolyte films for determination of cancer marker carbohydrate antigen 19-9. Microchim. Acta.

[56] Zhong X., Zhang M., Guo L., Xie Y., Luo R., Chen W., Cheng F., Wang L. (2021). A dual-signal self-checking photoelectrochemical immunosensor based on the sole composite of MIL-101(Cr) and CdSe quantum dots for the detection of alpha-fetoprotein. Biosens. Bioelectron..

[57] Atabaev T.S. (2018). Doped carbon dots for sensing and bioimaging applications: A minireview. Nanomaterials.

[58] Nekoueian K., Amiri M., Sillanpaa M., Marken F., Boukherroub R., Szunerits S. (2019). Carbon-based quantum particles: An electroanalytical and biomedical perspective. Chem. Soc. Rev..

[59] Wang Y.H., Huang K.J., Wu X. (2017). Recent advances in transition-metal dichalcogenides based electrochemical biosensors: A review. Biosens. Bioelectron..

[60] Muthusankar G., Rajkumar C., Chen S.-M., Karkuzhali R., Gopu G., Sangili A., Sengottuvelan N., Sankar R. (2019). Sonochemical driven simple preparation of nitrogen-doped carbon quantum dots/SnO_2_ nanocomposite: A novel electrocatalyst for sensitive voltammetric determination of riboflavin. Sens. Actuators B Chem..

[61] Muthusankar G., Devi R.K., Gopu G. (2020). Nitrogen-doped carbon quantum dots embedded Co_3_O_4_ with multiwall carbon nanotubes: An efficient probe for the simultaneous determination of anticancer and antibiotic drugs. Biosens. Bioelectron..

[62] Chowdhury A.D., Takemura K., Li T.C., Suzuki T., Park E.Y. (2019). Electrical pulse-induced electrochemical biosensor for hepatitis E virus detection. Nat. Commun..

[63] Roduner E. (2006). Size matters: Why nanomaterials are different. Chem. Soc. Rev..

[64] Jie G., Jie G. (2016). Sensitive electrochemiluminescence detection of cancer cells based on a CdSe/ZnS quantum dot nanocluster by multibranched hybridization chain reaction on gold nanoparticles. RSC Adv..

[65] Scatena R. (2015). Advances in Cancer Biomarkers: From Biochemistry to Clinic for a Critical Revision.

[66] Vaisocherova-Lisalova H., Visova I., Ermini M.L., Springer T., Song X.C., Mrazek J., Lamacova J., Scott Lynn N., Sedivak P., Homola J. (2016). Low-fouling surface plasmon resonance biosensor for multi-step detection of foodborne bacterial pathogens in complex food samples. Biosens. Bioelectron..

[67] Liu R., Zhang Y., Zhang S., Qiu W., Gao Y. (2013). Silver Enhancement of Gold Nanoparticles for Biosensing: From Qualitative to Quantitative. Appl. Spectrosc. Rev..

[68] Wan J., Ai J., Zhang Y., Geng X., Gao Q., Cheng Z. (2016). Signal-off impedimetric immunosensor for the detection of Escherichia coli O157:H7. Sci. Rep..

[69] Yang H., Xu Y., Hou Q., Xu Q., Ding C. (2022). Magnetic antifouling material based ratiometric electrochemical biosensor for the accurate detection of CEA in clinical serum. Biosens. Bioelectron..

[70] Zanoli L.M., D’Agata R., Spoto G. (2012). Functionalized gold nanoparticles for ultrasensitive DNA detection. Anal. Bioanal. Chem..

[71] Chen H., Song J., Li Y., Deng D., Song Y., Zhu X., Luo L. (2024). Cascade signal amplifying strategy for ultrasensitive detection of tumor biomarker by DNAzyme cleaving mediated HCR. Sens. Actuators B Chem..

[72] Upan J., Youngvises N., Tuantranont A., Karuwan C., Banet P., Aubert P.-H., Jakmunee J. (2021). A simple label-free electrochemical sensor for sensitive detection of alpha-fetoprotein based on specific aptamer immobilized platinum nanoparticles/carboxylated-graphene oxide. Sci. Rep..

[73] Fan X., Deng D., Chen Z., Qi J., Li Y., Han B., Luo L. (2021). A sensitive amperometric immunosensor for the detection of carcinoembryonic antigen using ZnMn_2_O_4_@reduced graphene oxide composites as signal amplifier. Sens. Actuators B Chem..

[74] Liu F., Chen H., Deng D., Fan X., Li Y., Madrakian T., Luo L. (2022). An ultrasensitive immunosensor based on cellulose nanofibrils/polydopamine/Cu-Ag nanocomposite for the detection of AFP. Bioelectrochemistry.

[75] Masud M.K., Na J., Younus M., Hossain M.S.A., Bando Y., Shiddiky M.J.A., Yamauchi Y. (2019). Superparamagnetic nanoarchitectures for disease-specific biomarker detection. Chem. Soc. Rev..

[76] Sharafeldin M., Bishop G.W., Bhakta S., El-Sawy A., Suib S.L., Rusling J.F. (2017). Fe_3_O_4_ nanoparticles on graphene oxide sheets for isolation and ultrasensitive amperometric detection of cancer biomarker proteins. Biosens. Bioelectron..

[77] Li Y., Wang Y., Zhang N., Fan D., Liu L., Yan T., Yang X., Ding C., Wei Q., Ju H. (2019). Magnetic electrode-based electrochemical immunosensor using amorphous bimetallic sulfides of CoSnSx as signal amplifier for the NT pro BNP detection. Biosens. Bioelectron..

[78] Zhang X., Bao N., Luo X., Ding S.N. (2018). Patchy gold coated Fe_3_O_4_ nanospheres with enhanced catalytic activity applied for paper-based bipolar electrode-electrochemiluminescence aptasensors. Biosens. Bioelectron..

[79] Shirazi H., Daneshpour M., Kashanian S., Omidfar K. (2015). Synthesis, characterization and in vitro biocompatibility study of Au/TMC/Fe_3_O_4_ nanocomposites as a promising, nontoxic system for biomedical applications. Beilstein J. Nanotechnol..

[80] Daneshpour M., Karimi B., Omidfar K. (2018). Simultaneous detection of gastric cancer-involved miR-106a and let-7a through a dual-signal-marked electrochemical nanobiosensor. Biosens. Bioelectron..

[81] Bai K.P., Zhou L.J., Yang G.P., Cao M.X., Wang Y.Y. (2020). Four new metal-organic frameworks based on diverse metal clusters: Syntheses, structures, luminescent sensing and dye adsorption properties. J. Solid State Chem..

[82] Mohan B., Modi K., Patel C., Bhatia P., Kumar P., Kumar A., Sharma H.K. (2018). Selectivity for La^3+^ ion by synthesized 4-((5-methylfuran-2-yl)methylene)hydrazono)methyl)phenol receptor and its spectral analysis. Spectrochim. Acta Part A Mol. Biomol. Spectrosc..

[83] Mohan B., Kumar S., Xi H., Ma S., Tao Z., Xing T., You H., Zhang Y., Ren P. (2022). Fabricated Metal-organic frameworks (MOFs) as luminescent and electrochemical biosensors for cancer biomarkers detection. Biosens. Bioelectron..

[84] Fu L., Yang Z., Wang Y., Li R., Zhai J. (2021). Construction of Metal-organic frameworks (MOFs)–based membranes and their ion transport applications. Small Sci..

[85] Fu X., He J., Zhang C., Chen J., Wen Y., Li J., Mao W., Zhong H., Wu J., Ji X. (2019). Trimetallic signal amplification aptasensor for TSP-1 detection based on Ce-MOF@Au and AuPtRu nanocomposites. Biosens. Bioelectron..

[86] Li X., Li X., Li D., Zhao M., Wu H., Shen B., Liu P., Ding S. (2020). Electrochemical biosensor for ultrasensitive exosomal miRNA analysis by cascade primer exchange reaction and MOF@Pt@MOF nanozyme. Biosens. Bioelectron..

[87] Hatami Z., Jalali F., Amouzadeh Tabrizi M., Shamsipur M. (2019). Application of metal-organic framework as redox probe in an electrochemical aptasensor for sensitive detection of MUC1. Biosens. Bioelectron..

[88] Zhang Y., Vaccarella S., Morgan E., Li M., Etxeberria J., Chokunonga E., Manraj S.S., Kamate B., Omonisi A., Bray F. (2023). Global variations in lung cancer incidence by histological subtype in 2020: A population-based study. Lancet Oncol..

[89] Tang J., Liu L., Qin J., Lv X., Li J., Tang D., Zhuang J. (2022). Biocatalysis-mediated MOF-to-prussian blue transformation enabling sensitive detection of NSCLC-associated miRNAs with dual-readout signals. Biosens. Bioelectron..

[90] Dezhakam E., Vayghan R.F., Dehghani S. (2024). Highly efficient electrochemical biosensing platform in breast cancer detection based on MOF-COF@Au core-shell like nanostructure. Sci. Rep..

[91] Ferlay J., Soerjomataram I., Dikshit R., Eser S., Mathers C., Rebelo M., Parkin D.M., Forman D., Bray F. (2015). Cancer incidence and mortality worldwide: Sources, methods and major patterns in GLOBOCAN 2012. Int. J. Cancer.

[92] Zhou J., Lin Q., Huang Z., Xiong H., Yang B., Chen H., Kong J. (2022). Aptamer-initiated catalytic hairpin assembly fluorescence assay for universal, sensitive exosome detection. Anal. Chem..

[93] Gong Y., Ji P., Yang Y.S., Xie S., Yu T.J., Xiao Y., Jin M.L., Ma D., Guo L.W., Pei Y.C. (2021). Metabolic-pathway-based subtyping of triple-negative breast cancer reveals potential therapeutic targets. Cell Metab..

[94] Britt K.L., Cuzick J., Phillips K.A. (2020). Key steps for effective breast cancer prevention. Nat. Rev. Cancer.

[95] Wang X., Huang J., Chen W., Li G., Li Z., Lei J. (2022). The updated role of exosomal proteins in the diagnosis, prognosis, and treatment of cancer. Exp. Mol. Med..

[96] Hannafon B., Ding W.Q. (2013). Intercellular communication by exosome-derived microRNAs in cancer. Int. J. Mol. Sci..

[97] Dervisevic M., Alba M., Adams T.E., Prieto-Simon B., Voelcker N.H. (2021). Electrochemical immunosensor for breast cancer biomarker detection using high-density silicon microneedle array. Biosens. Bioelectron..

[98] Carvajal S., Fera S.N., Jones A.L., Baldo T.A., Mosa I.M., Rusling J.F., Krause C.E. (2018). Disposable inkjet-printed electrochemical platform for detection of clinically relevant HER-2 breast cancer biomarker. Biosens. Bioelectron..

[99] Xu Y., Zhang Y., Li N., Yang M., Xiang T., Huo D., Qiu Z., Yang L., Hou C. (2022). An ultra-sensitive dual-signal ratiometric electrochemical aptasensor based on functionalized MOFs for detection of HER2. Bioelectrochemistry.

[100] Wang Y., Wang Y., Chen G., Li Y., Xu W., Gong S. (2017). Quantum-dot-based theranostic micelles conjugated with an anti-EGFR nanobody for triple-negative breast cancer therapy. ACS Appl. Mater. Interfaces.

[101] Wang M., Liu W., Zhang Y., Dang M., Zhang Y., Tao J., Chen K., Peng X., Teng Z. (2019). Intercellular adhesion molecule 1 antibody-mediated mesoporous drug delivery system for targeted treatment of triple-negative breast cancer. J. Colloid Interface Sci..

[102] Zhang M., Xia L., Mei W., Zou Q., Liu H., Wang H., Zou L., Wang Q., Yang X., Wang K. (2023). One-step multiplex analysis of breast cancer exosomes using an electrochemical strategy assisted by gold nanoparticles. Anal. Chim. Acta.

[103] Han B., Sha L., Yu X., Yang M., Cao Y., Zhao J. (2021). Identification of dual therapeutic targets assisted by in situ automatous DNA assembly for combined therapy in breast cancer. Biosens. Bioelectron..

[104] He L., Hannon G.J. (2004). MicroRNAs: Small RNAs with a big role in gene regulation. Nat. Rev. Genet..

[105] Curtale G., Mirolo M., Renzi T.A., Rossato M., Bazzoni F., Locati M. (2013). Negative regulation of Toll-like receptor 4 signaling by IL-10-dependent microRNA-146b. Proc. Natl. Acad. Sci. USA.

[106] Drury R.E., O’Connor D., Pollard A.J. (2017). The clinical application of microRNAs in infectious disease. Front. Immunol..

[107] Chinnappan M., Singh A.K., Kakumani P.K., Kumar G., Rooge S.B., Kumari A., Varshney A., Rastogi A., Singh A.K., Sarin S.K. (2014). Key elements of the RNAi pathway are regulated by hepatitis B virus replication and HBx acts as a viral suppressor of RNA silencing. Biochem. J..

[108] Fu Y.R., Liu X.J., Li X.J., Shen Z.Z., Yang B., Wu C.C., Li J.F., Miao L.F., Ye H.Q., Qiao G.H. (2015). MicroRNA miR-21 attenuates human cytomegalovirus replication in neural cells by targeting Cdc25a. J. Virol..

[109] Ondevilla N.A.P., Liu P.-W., Huang W.-T., Weng T.-P., Lee N.-Y., Ma S.-C., Huang J.-J., Wong T.-W., Chang H.-C. (2024). A point-of-care electrochemical biosensor for the rapid and sensitive detection of biomarkers in murine models with LPS-induced sepsis. Biosens. Bioelectron..

[110] Hannafon B.N., Trigoso Y.D., Calloway C.L., Zhao Y.D., Lum D.H., Welm A.L., Zhao Z.J., Blick K.E., Dooley W.C., Ding W.Q. (2016). Plasma exosome microRNAs are indicative of breast cancer. Breast Cancer Res..

[111] Cao Y., Yu X., Zeng T., Fu Z., Zhao Y., Nie B., Zhao J., Yin Y., Li G. (2022). Molecular characterization of exosomes for subtype-based diagnosis of breast cancer. J. Am. Chem. Soc..

[112] Cheng W., Yao Y., Li D., Duan C., Wang Z., Xiang Y. (2023). Asymmetrically split DNAzyme-based colorimetric and electrochemical dual-modal biosensor for detection of breast cancer exosomal surface proteins. Biosens. Bioelectron..

[113] Moura S.L., Martin C.G., Marti M., Pividori M.I. (2020). Electrochemical immunosensing of nanovesicles as biomarkers for breast cancer. Biosens. Bioelectron..

[114] Cohen P.A., Jhingran A., Oaknin A., Denny L. (2019). Cervical cancer. Lancet.

[115] Bray F., Ferlay J., Soerjomataram I., Siegel R.L., Torre L.A., Jemal A. (2018). Global cancer statistics 2018: GLOBOCAN estimates of incidence and mortality worldwide for 36 cancers in 185 countries. A Cancer J. Clin..

[116] Clifford G., Franceschi S., Diaz M., Munoz N., Villa L.L. (2006). Chapter 3: HPV type-distribution in women with and without cervical neoplastic diseases. Vaccine.

[117] Crosbie E.J., Einstein M.H., Franceschi S., Kitchener H.C. (2013). Human papillomavirus and cervical cancer. Lancet.

[118] Yang N., Liu P., Cai C., Zhang R., Sang K., Shen P., Huang Y., Lu Y. (2021). Triple signal amplification strategy for the ultrasensitive electrochemical detection of human papillomavirus 16 E6/E7 mRNA. Enzym. Microb. Technol..

[119] Teengam P., Siangproh W., Tuantranont A., Henry C.S., Vilaivan T., Chailapakul O. (2017). Electrochemical paper-based peptide nucleic acid biosensor for detecting human papillomavirus. Anal. Chim. Acta.

[120] Jampasa S., Wonsawat W., Rodthongkum N., Siangproh W., Yanatatsaneejit P., Vilaivan T., Chailapakul O. (2014). Electrochemical detection of human papillomavirus DNA type 16 using a pyrrolidinyl peptide nucleic acid probe immobilized on screen-printed carbon electrodes. Biosens. Bioelectron..

[121] Mahmoodi P., Rezayi M., Rasouli E., Avan A., Gholami M., Ghayour Mobarhan M., Karimi E., Alias Y. (2020). Early-stage cervical cancer diagnosis based on an ultra-sensitive electrochemical DNA nanobiosensor for HPV-18 detection in real samples. J. Nanobiotechnol..

[122] Koo Siew Kim N.S., Parmin N.A., Hashim U., Gopinath S.C.B., Rejali Z., Afzan A., Uda M.N.A., Afnan Uda M.N., Hong V.C. (2020). Electrochemical DNA biosensor based on 30 nM gold nanoparticle modified electrode by electroless deposition for human papillomavirus (HPV) 18 E6 region. IOP Conf. Ser. Mater. Sci. Eng..

[123] Chaibun T., Thanasapburachot P., Chatchawal P., Su Yin L., Jiaranuchart S., Jearanaikoon P., Promptmas C., Buajeeb W., Lertanantawong B. (2022). A multianalyte electrochemical genosensor for the detection of high-risk HPV genotypes in oral and cervical cancers. Biosensors.

[124] Civit L., Fragoso A., Holters S., Durst M., O’Sullivan C.K. (2012). Electrochemical genosensor array for the simultaneous detection of multiple high-risk human papillomavirus sequences in clinical samples. Anal. Chim. Acta.

[125] Siegel R.L., Giaquinto A.N., Jemal A. (2024). Cancer statistics, 2024. A Cancer J. Clin..

[126] Yang G., Xiao Z., Tang C., Deng Y., Huang H., He Z. (2019). Recent advances in biosensor for detection of lung cancer biomarkers. Biosens. Bioelectron..

[127] Mahmud N., Anik M.I., Hossain M.K., Khan M.I., Uddin S., Ashrafuzzaman M., Rahaman M.M. (2022). Advances in nanomaterial-based platforms to combat COVID-19: Diagnostics, preventions, therapeutics, and vaccine developments. ACS Appl. Bio Mater..

[128] Arya S.K., Bhansali S. (2011). Lung cancer and its early detection using biomarker-based biosensors. Chem. Rev..

[129] Mir T.A., Yoon J.H., Gurudatt N.G., Won M.S., Shim Y.B. (2015). Ultrasensitive cytosensing based on an aptamer modified nanobiosensor with a bioconjugate: Detection of human non-small-cell lung cancer cells. Biosens. Bioelectron..

[130] Reckamp K.L., Melnikova V.O., Karlovich C., Sequist L.V., Camidge D.R., Wakelee H., Perol M., Oxnard G.R., Kosco K., Croucher P. (2016). A highly sensitive and quantitative test platform for detection of NSCLC EGFR mutations in urine and plasma. J. Thorac. Oncol..

[131] Shoja Y., Kermanpur A., Karimzadeh F. (2018). Diagnosis of EGFR exon21 L858R point mutation as lung cancer biomarker by electrochemical DNA biosensor based on reduced graphene oxide/functionalized ordered mesoporous carbon/Ni-oxytetracycline metallopolymer nanoparticles modified pencil graphite electrode. Biosens. Bioelectron..

[132] Shin Low S., Pan Y., Ji D., Li Y., Lu Y., He Y., Chen Q., Liu Q. (2020). Smartphone-based portable electrochemical biosensing system for detection of circulating microRNA-21 in saliva as a proof-of-concept. Sens. Actuators B Chem..

[133] Wu T., Chen W., Kong D., Li X., Lu H., Liu S., Wang J., Du L., Kong Q., Huang X. (2015). miR-25 targets the modulator of apoptosis 1 gene in lung cancer. Carcinogenesis.

[134] Han K., Liu H., Cui J., Liu Y., Pan P. (2023). Recent strategies for electrochemical sensing detection of miRNAs in lung cancer. Anal. Biochem..

[135] Sheng Y., Zhang T., Zhang S., Johnston M., Zheng X., Shan Y., Liu T., Huang Z., Qian F., Xie Z. (2021). A CRISPR/Cas13a-powered catalytic electrochemical biosensor for successive and highly sensitive RNA diagnostics. Biosens. Bioelectron..

[136] Asadzadeh-Firouzabadi A., Zare H.R. (2018). Preparation and application of AgNPs/SWCNTs nanohybrid as an electroactive label for sensitive detection of miRNA related to lung cancer. Sens. Actuators B Chem..

[137] Gao W., Xu J., Liu L., Shen H., Zeng H., Shu Y. (2012). A systematic-analysis of predicted miR-21 targets identifies a signature for lung cancer. Biomed. Pharmacother..

[138] Zhou X., Liu H., Pang Y., Wang M., Liu S. (2022). UTMD-mediated delivery of miR-21-5p inhibitor suppresses the development of lung cancer. Tissue Cell.

[139] Liu S., Su W., Li Y., Zhang L., Ding X. (2018). Manufacturing of an electrochemical biosensing platform based on hybrid DNA hydrogel: Taking lung cancer-specific miR-21 as an example. Biosens. Bioelectron..

[140] Anagnostou V.K., Syrigos K.N., Bepler G., Homer R.J., Rimm D.L. (2009). Thyroid transcription factor 1 is an independent prognostic factor for patients with stage I lung adenocarcinoma. J. Clin. Oncol..

[141] Rekhtman N., Ang D.C., Sima C.S., Travis W.D., Moreira A.L. (2011). Immunohistochemical algorithm for differentiation of lung adenocarcinoma and squamous cell carcinoma based on large series of whole-tissue sections with validation in small specimens. Mod. Pathol..

[142] Gloriane C.L.H., Severino Imasa M., Juat N., Hernandez K.V., May Sayo T., Cristal-Luna G., Marie Asur-Galang S., Bellengan M., John Duga K., Brian Buenaobra B. (2023). Expression landscapes in non-small cell lung cancer shaped by the thyroid transcription factor 1. Lung Cancer.

[143] Wang W., Tang H., Zhou L., Li Z. (2024). A novel label-free electrochemical immunosensor for the detection of thyroid transcription factor 1 using ribbon-like tungsten disulfide-reduced graphene oxide nanohybrids and gold nanoparticles. Molecules.

[144] Liu C., Shen X., Yan L., Qu R., Wang Y., He Y., Zhan Z., Chen P., Lin F. (2024). Controllable self-assembled DNA nanomachine enable homogeneous rapid electrochemical one-pot assay of lung cancer circulating tumor cells. Biosens. Bioelectron..

[145] Wu C., Yan L., Zhan Z., Qu R., Wang Y., Zeng X., Yang H., Feng P., Wei Z., Chen P. (2024). Biomolecules-mediated electrochemical signals of Cu^2+^: Y-DNA nanomachines enable homogeneous rapid one-step assay of lung cancer circulating tumor cells. Biosens. Bioelectron..

[146] Megyesfalvi Z., Gay C.M., Popper H., Pirker R., Ostoros G., Heeke S., Lang C., Hoetzenecker K., Schwendenwein A., Boettiger K. (2023). Clinical insights into small cell lung cancer: Tumor heterogeneity, diagnosis, therapy, and future directions. A Cancer J. Clin..

[147] Gazdar A.F., Bunn P.A., Minna J.D. (2017). Small-cell lung cancer: What we know, what we need to know and the path forward. Nat. Rev. Cancer.

[148] Gay C.M., Stewart C.A., Park E.M., Diao L., Groves S.M., Heeke S., Nabet B.Y., Fujimoto J., Solis L.M., Lu W. (2021). Patterns of transcription factor programs and immune pathway activation define four major subtypes of SCLC with distinct therapeutic vulnerabilities. Cancer Cell.

[149] Haque A., Polcyn R., Matzelle D., Banik N.L. (2018). New insights into the role of neuron-specific enolase in neuro-inflammation, neurodegeneration, and neuroprotection. Brain Sci..

[150] Karaman C., Bölükbaşı O.S., Yola B.B. (2022). Electrochemical neuron-specific enolase (NSE) immunosensor based on CoFe_2_O_4_@Ag nanocomposite and AuNPs@MoS_2_/rGO. Anal. Chim. Acta.

[151] Alberts S.R., Cervantes A., van de Velde C.J. (2003). Gastric cancer: Epidemiology, pathology and treatment. Ann. Oncol..

[152] Joshi S.S., Badgwell B.D. (2021). Current treatment and recent progress in gastric cancer. A Cancer J. Clin..

[153] Fock K.M. (2014). Review article: The epidemiology and prevention of gastric cancer. Aliment. Pharmacol. Ther..

[154] Luo K., Zhao C., Luo Y., Pan C., Li J. (2022). Electrochemical sensor for the simultaneous detection of CA72-4 and CA19-9 tumor markers using dual recognition via glycosyl imprinting and lectin-specific binding for accurate diagnosis of gastric cancer. Biosens. Bioelectron..

[155] Hashimoto I., Oshima T. (2022). Claudins and gastric cancer: An overview. Cancers.

[156] Kanagavalli P., Eissa S. (2024). Exploring various carbon nanomaterials-based electrodes modified with polymelamine for the reagentless electrochemical immunosensing of Claudin18.2. Biosens. Bioelectron..

[157] Cho E.J., Kim H.K., Jeong T.D., Ko D.H., Bae S.E., Lee J.S., Lee W., Choe J.W., Chun S., Jung H.Y. (2016). Method evaluation of pepsinogen I/II assay based on chemiluminescent immunoassays and comparison with other test methods. Clin. Chim. Acta.

[158] Mansour-Ghanaei F., Joukar F., Baghaee M., Sepehrimanesh M., Hojati A. (2019). Only serum pepsinogen I and pepsinogen I/II ratio are specific and sensitive biomarkers for screening of gastric cancer. Biomolelar Concepts.

[159] Kanagavalli P., Eissa S. (2024). Redox probe-free electrochemical immunosensor utilizing electropolymerized melamine on reduced graphene oxide for the point-of-care diagnosis of gastric cancer. Talanta.

[160] Huang R., He L., Xia Y., Xu H., Liu C., Xie H., Wang S., Peng L., Liu Y., Liu Y. (2019). A sensitive aptasensor based on a hemin/G-quadruplex-assisted signal amplification strategy for electrochemical detection of gastric cancer exosomes. Small.

[161] Tsai M.M., Wang C.S., Tsai C.Y., Huang H.W., Chi H.C., Lin Y.H., Lu P.H., Lin K.H. (2016). Potential diagnostic, prognostic and therapeutic targets of microRNAs in human gastric cancer. Int. J. Mol. Sci..

[162] Oo S.L., Venkatesh S., Karthikeyan V., Arava C.M., Pathikonda S., Yu P.K.N., Lau T.C.K., Chen X., Roy V.A.L. (2021). Highly sensitive and cost-effective portable sensor for early gastric carcinoma diagnosis. Sensors.

[163] Zhuang J., Wan H., Zhang X. (2021). Electrochemical detection of miRNA-100 in the sera of gastric cancer patients based on DSN-assisted amplification. Talanta.

[164] Zhu H., Chen S., Lan F., Li W., Ji T., Zhang L., Guo Y., Pan W., Luo S., Xie R. (2024). Sensitive electrochemical biosensor for rapid detection of sEV-miRNA based turbo-like localized catalytic hairpin assembly. Anal. Chim. Acta.

[165] Saputra H.A., Karim M.M. (2025). Fundamentals and research progression on electrochemical sensing of colorectal cancer. Microchim. Acta.

[166] Norouzi S., Alipour E., Soltani S., Hallaj-Nezhadi S., Hashemzadeh S. (2025). A novel electrochemical biosensor with liposomal amplification for sensitive detection of colon cancer biomarker: A step toward early cancer diagnosis. Talanta.

[167] Chen Y., Ye Z., Ma M., Yang J., Liu R., Zhang Y., Ma P., Song D. (2024). Electrochemiluminescence biosensor for specific detection of pancreatic ductal carcinoma through dual targeting of MUC1 and miRNA-196a. Biosens. Bioelectron..

[168] Huynh T.V., Tran H.L., Anh N.T.N., Doong R.A. (2024). Electrochemical sensor for rapid diagnosis and early-stage detection of pancreatic cancer using Er-GQDs decorated MoS_2_ nanoflowers. Sens. Actuators B Chem..

[169] Dehghani P., Salehirozveh M., Tajabadi A., Yeung C.C., Lam M., Leung H.Y., Roy V.A.L. (2025). Next-gen point-of-care tool for ultra-sensitive detection of urinary spermine for prostate cancer diagnosis. ACS Sensors.

[170] Sadeghi M., Ehzari H., Ghasemi Y. (2025). Ultrahighly sensitive sandwich-type electrochemical immunosensor for selective detection of prostate specific antigen based on functionalized bimetallic Au-Ag nanoclusters as label for signal amplification. Microchem. J..

[171] Oyowvi M.O., Babawale K.H., Atere A.D. (2025). Emerging nanotechnologies and their role in early ovarian cancer detection, diagnosis and interventions. J. Ovarian Res..

[172] Sim S., Wong N.K. (2021). Nanotechnology and its use in imaging and drug delivery. Biomed. Rep..

[173] Kivrak E., Kara P. (2025). Simultaneous detection of ovarian cancer related miRNA biomarkers with carboxylated graphene oxide modified electrochemical biosensor platform. Bioelectrochemistry.

